# Metabolomics and Cardiovascular Risk in Patients with Heart Failure: A Systematic Review and Meta-Analysis

**DOI:** 10.3390/ijms25115693

**Published:** 2024-05-23

**Authors:** Leonel Sousa Neves, Francisca Saraiva, Rita Ferreira, Adelino Leite-Moreira, António S. Barros, Sílvia O. Diaz

**Affiliations:** 1 Cardiovascular R&D Centre, UnIC@RISE, Department of Surgery and Physiology, Faculty of Medicine, University of Porto, 4200-319 Porto, Portugal; sousanevesleonel@gmail.com (L.S.N.); f.saraiva@med.up.pt (F.S.); amoreira@med.up.pt (A.L.-M.); 2 LAQV-REQUIMTE, Department of Chemistry, University of Aveiro, 3810-193 Aveiro, Portugal; ritaferreira@ua.pt

**Keywords:** biomarkers, cardiovascular disease, heart failure, metabolomics, prognosis

## Abstract

The associations of plasma metabolites with adverse cardiovascular (CV) outcomes are still underexplored and may be useful in CV risk stratification. We performed a systematic review and meta-analysis to establish correlations between blood metabolites and adverse CV outcomes in patients with heart failure (HF). Four cohorts were included, involving 83 metabolites and 37 metabolite ratios, measured in 1158 HF patients. Hazard ratios (HR) of 42 metabolites and 3 metabolite ratios, present in at least two studies, were combined through meta-analysis. Higher levels of histidine (HR 0.74, 95% CI [0.64; 0.86]) and tryptophan (HR 0.82 [0.71; 0.96]) seemed protective, whereas higher levels of symmetric dimethylarginine (SDMA) (HR 1.58 [1.30; 1.93]), N-methyl-1-histidine (HR 1.56 [1.27; 1.90]), SDMA/arginine (HR 1.38 [1.14; 1.68]), putrescine (HR 1.31 [1.06; 1.61]), methionine sulfoxide (HR 1.26 [1.03; 1.52]), and 5-hydroxylysine (HR 1.25 [1.05; 1.48]) were associated with a higher risk of CV events. Our findings corroborate important associations between metabolic imbalances and a higher risk of CV events in HF patients. However, the lack of standardization and data reporting hampered the comparison of a higher number of studies. In a future clinical scenario, metabolomics will greatly benefit from harmonizing sample handling, data analysis, reporting, and sharing.

## 1. Introduction

Heart failure (HF) is a complex clinical syndrome caused by structural and/or functional heart abnormalities, resulting in elevated intracardiac pressures and/or inadequate cardiac output [1]. The prevalence of HF is estimated to be 1–2% of the overall adult population [2], affecting over 10% of those aged over 70 years and with a rising incidence [1]. Patients diagnosed with HF have poor prognosis, with an associated 1-year mortality risk of 15–30% and 1-year hospital readmission risk of 50% [3].

Currently, the assessment and monitoring of HF patients consists of the combined evaluation of clinical signs and symptoms, echocardiography, and blood natriuretic peptides (namely B-type natriuretic peptide—BNP or N-terminal pro-B-type natriuretic peptide—NT-proBNP) [1,4], whose levels are known to be associated with prognosis [5]. While many other biomarkers have been proposed, such as markers of myocardial injury (cardiac troponins T and I) [6], inflammation and oxidative stress (interleukin-6 and tumor necrosis factor alfa) [7], vascular dysfunction (endothelin-1) [8], and matrix remodeling (matrix metalloproteinase-2) [9], and shown to provide incremental prognostic value over natriuretic peptides, there is no evidence of their incremental benefit in HF management [10,11].

Metabolomics is a valuable approach to uncover the molecular processes and biological pathways affected in pathological states, thus contributing to the understanding of complex multifactorial diseases [12], as is the case with HF. Hence, it is a conceivable strategy to explore the onset of metabolic dysregulations underlying CV events [12]. Given the multifactorial nature of HF, often diagnosed in older patients with other underlying comorbidities [1], metabolomics may provide important insights into the disease pathogenesis as well as to identify putative biomarkers associated with patient’s clinical trajectories and outcomes [12,13]. The most prominent advances in metabolomics in HF have suggested that HF severity and prognosis may be reflected in the plasma metabolome [14,15]. In fact, a poor prognosis of HF patients has been associated with changes in circulating ceramides, amino acids, acylcarnitines, and organic acids, which broadly indicate a switch in energy and amino acids metabolism [14,15]. Some studies have proposed different metabolite-based profiles such as the Prognostic Metabolic Profile (PMP) or the Cardiac Lipid Panel (CLP) [16,17], the latter showing promising results compared to other clinical scores [17]. However, none of the metabolite-based scores have reached clinical application, due to the lack of robust comparison with scores already in clinical use.

Despite significant efforts to identify blood metabolites with prognostic value, there is no systematic, quantitative, or qualitative compilation of the current knowledge in the HF population. Therefore, this meta-analysis aimed to gather risk associations between blood metabolites and CV outcomes in patients with HF.

## 2. Methods

The systematic review was carried out following the Preferred Reporting Items for Systematic Reviews and Meta-Analyses (PRISMA) checklist (Table S5) [18]. This review protocol was not registered in any platform.

### 2.1. Search Strategy

We performed an electronic search of the PubMed and ISI Web of Knowledge databases (last searched on 31 December 2022) to identify all articles related to the association of individual blood metabolites and CV outcomes in HF patients. The query string “(Metabolomics OR Lipidomics) AND heart failure” (with “Humans” filter in Pubmed) was used for the search. In the ISI Web of Knowledge database, the same query string was used with the exclusion of the “Animal”, “Animals”, “Mouse”, “mice”, “rats”, and “review” keywords.

### 2.2. Eligibility Criteria

In this study, we only included original publications without any language restrictions. Reviews and meta-analyses, comments, guidelines, editorials or letters, conference summaries, and non-longitudinal studies were excluded. From the results of the initial query, we filtered based on the following inclusion criteria: (1) studies that considered adult (>18 years old) patients diagnosed with HF; (2) studies that leveraged targeted or untargeted metabolomics; (3) studies that used blood samples as plasma or serum; and (4) studies that reported the time-to-event adjusted association between individual metabolites and major outcomes such as all-cause death or all-cause hospitalization. We excluded articles that reported metabolites not measured in at least two cohorts.

### 2.3. Screening

**Figure 1:**
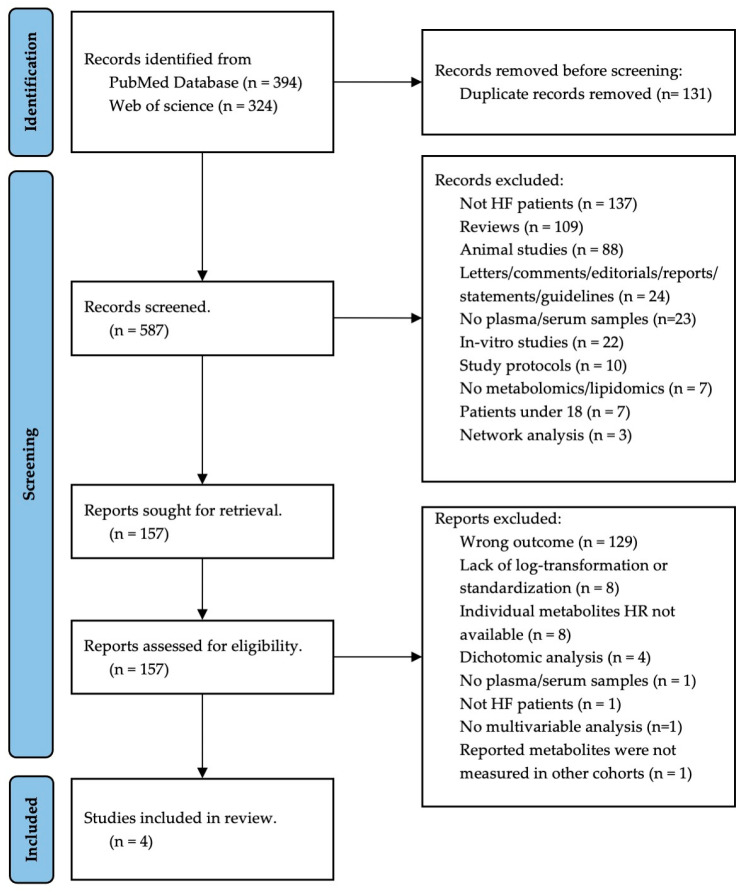
Prisma 2020 flow diagram.

LSN screened all the article titles and abstracts. Disagreements were resolved by consensus among the LSN, SOD, and ASB. Full texts of articles deemed potentially eligible during the initial screening were obtained for further reading. The search and selection processes can be found in the PRISMA flow diagram (Figure 1).

### 2.4. Data Collection Process and Data Items

Data were extracted in a standardized form into a Microsoft Excel^®^ spreadsheet by LSN and confirmed by SOD. Clinical and methodological characteristics were collected from all included studies: study characteristics (author, year), study design, participant characteristics, cohort name, sample size, analytical platform, metabolomics approach (targeted or untargeted), type of blood sample (serum or plasma), mean or median follow-up time, data preprocessing (e.g., log-transformation, standardization), outcomes assessed, number of events and variables used for adjustment. For each metabolite, adjusted hazard ratios (HR) and 95% confidence intervals (95% CI) were retrieved. Metabolite classes and subclasses were also gathered from the Human Metabolome Database and were included in the database [19].

### 2.5. Data Aggregation Approach

Metabolites were included in the meta-analysis if they were measured in at least 2 studies and if the authors explicitly reported that their levels were log-transformed and standardized. The latter step was used to ensure that the HR values were comparable, thus reducing the bias inherent to the experimental characteristics. Given the small number of publications available, no subgroup or sensitivity analyses were performed.

### 2.6. Risk of Bias

The quality of observational included studies was assessed using the Newcastle–Ottawa Scale [20], maximum of nine stars (Table S3), by LSN, SOD, and FS.

### 2.7. Data Analysis

Continuous variables are shown as means (standard deviations) or medians (interquartile ranges), as reported by the authors in the original publications. Meta-analysis was performed using random-effects models to compute combined statistical measures (HR) and 95% confidence intervals (CI). We chose to use the random-effects model, as it incorporates both within- and between-study variance components [21]. Random-effects models account for the variability among study results beyond chance, using the DerSimonian and Laird method. The choice of this model was made based on the clinical and methodological diversity across studies, which could influence the metabolite levels in HF patients.

For each meta-analytical measure, the *I*^2^, which measures the percentage of total variation across studies due to heterogeneity rather than chance, was also calculated. Values of *I*^2^ greater than 50% were considered indicative of substantial heterogeneity, while *I*^2^ lower than 50% were considered low to moderate [21].

Calculations were carried out in R Statistical Software R [22], version 4.1.12, along with the *meta* package [23], using the metagen() command for combining HR and calculating overall effect and CI.

## 3. Results

### 3.1. Study Selection

The flow diagram of the study is shown in Figure 1. In the original search, 718 entries were found, all of which were published from 2005 to 2022, 131 were duplicated. The remaining 587 records were screened by title and abstract, of which 157 were retrieved and their eligibility was assessed through full-text analysis. Out of these, seven studies met all the inclusion criteria except for the data preprocessing criteria (log-transformation and standardization) [17,24,25,26,27,28,29]. One article met all the inclusion criteria, but the metabolite measured was not measured in any other cohort [30]. A total of four articles were eligible and sought for meta-analysis (Table 1) [31,32,33,34].

### 3.2. Study Endpoints

Two studies considered all-cause mortality outcome [32,33], one study explored a composite outcome of all-cause death and unscheduled readmission due to worsening HF or lethal arrhythmia [34], and the last one defined the study outcome as all-cause mortality or heart failure hospitalization [31] (see Table 1).

### 3.3. Patients’ Characteristics

Patient characteristics gathered from the original publications are summarized in Table S1. 

The selected studies included 4 cohorts [31,32,33,34], with a total of 1158 patients. The minimal number of patients was 138 [31], and the maximum was 479 [33]. The included studies reported the HR for a total of 83 metabolites and 37 metabolite ratios (Table S2), of which 42 metabolites and 3 ratios were reported in at least 2 cohorts. Follow-up times were reported as mean [34] or median [31,32,33], and ranged from 1 year [34] to 6.3 years [33]. The prevalence of events (calculated as the percentage of events within each N) ranged from 13% [34] to 38% [31].

All four cohorts included patients diagnosed with HF, although some differences should be acknowledged (Table 1). Du et al. included patients with acute heart failure (AHF) after primary percutaneous coronary intervention for ST-segment elevation myocardial infarction treatment [31]. Kouzu et al. included both acute and chronic HF patients [34], and all cohorts explored by Zhang et al. included patients with HF with reduced ejection fraction (HFrEF) [32,33].

### 3.4. Metabolomics Characteristics

Included studies were targeted for amino acids [31,34], for amino acids and biogenic amines [32], or for oxylipins [33]. Three studies were based on MS platforms, coupled with either ultra-performance liquid chromatography (UPLC) [32] or liquid chromatography (LC) [31,33]. Kouzu et al. used only a UPLC-based platform [34]. Two studies used serum samples [32,33] and the other two used plasma samples [31,34].

### 3.5. Explored Metabolites

The 42 metabolites included in the meta-analysis belonged to 6 classes and 7 subclasses according to the human metabolome database (Table S2) [19], namely 1 tryptamine, 2 amines, 35 amino acids or analogs, 1 carbonyl compound, 1 indolyl carboxylic acid, 1 organic sulfonic acid, and 1 phosphate ester. 

### 3.6. Risk of Bias in Studies

The Newcastle–Ottawa Scale confirmed the good quality of all included studies (Table S3), with a minimum of seven [34] and a maximum of nine stars [31] (maximum range of scale grade is nine).

### 3.7. Data Pre-Processing

Preprocessing of data is a critical step, impacting the robustness of the findings (i.e., the amount of biologically relevant information within the study) and the comparability of results (between studies) [35]. We defined the use of log-transformation and standardization (to overcome skewness and heteroscedasticity) as an inclusion criterion to ensure the comparability of the HR. Thus, the associations between outcomes and metabolites, in this case HR, refer to a 1-SD change in the log-transformed metabolite range, minimizing differences inherent to the equipment/laboratory used (i.e., sensitivity or limits of detection).

We observed a significant dispersion in data preprocessing. Some studies met all the inclusion criteria but refrained from employing any form of pretreatment or transformation [25,26,28,29], whereas others exclusively transformed the metabolite data [17,24,27]. Additionally, one study only pre-processed the data concerning a single metabolite instead of the whole dataset [34]. This lack of standardization in data analysis and reporting significantly limited the number of studies that could be combined through meta-analysis.

### 3.8. Primary Analysis

**Figure 2:**
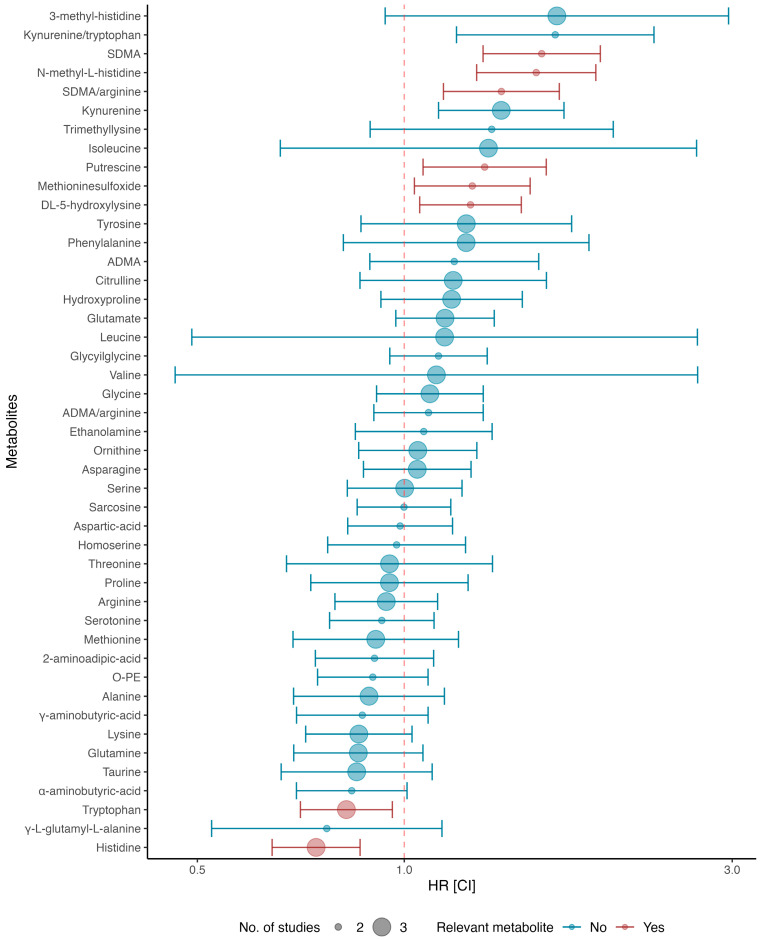
Forest plot of the random-effects computed HR. Red marks indicate relevant associated metabolites.

Of the 42 metabolites and 3 ratios explored, the meta-analysis showed that 7 metabolites and 1 metabolite ratio were relevantly associated (HR and 95% CI > 1 or <1, *I*^2^ < 50%) with the cardiovascular outcome (Figure 2 and Table S4). Higher histidine (three studies, pooled HR 0.74, 95% CI [0.64; 0.86], *I*^2^: 0%, *p*-value = 0.44) and tryptophan (three studies, pooled HR 0.82 [0.71; 0.96], *I*^2^: 0%, *p*-value = 0.51) levels were associated with a lower risk of CV events, whereas higher symmetric dimethylarginine (SDMA) (two studies, pooled HR 1.58 [1.30; 1.93], *I*^2^: 0%, *p*-value = 0.41), N-methyl-1-histidine (two studies, pooled HR 1.56 [1.27; 1.90] *I*^2^: 0%, *p*-value = 0.56), SDMA/arginine (two studies, pooled HR 1.38 [1.14; 1.68], *I*^2^: 0%, *p*-value = 0.48), putrescine (two studies, pooled HR 1.31 [1.06; 1.61], *I*^2^: 0%, *p*-value = 0.81), methionine sulfoxide (two studies, pooled HR 1.26 [1.03; 1.52], *I*^2^: 0%, *p*-value = 0.88), and 5-hydroxylysine (two studies, pooled HR 1.25 [1.05; 1.48], *I*^2^: 0%, *p*-value = 0.97) levels were associated with a higher risk of CV events. Kynurenine (three studies, pooled HR 1.38 [1.12; 1.71], *I*^2^: 51.4%, *p*-value = 0.13 and the kynurenine/tryptophan ratio (two studies, pooled HR 1.66 [1.19; 2.31], *I*^2^:71%, *p*-value = 0.06) were statistically associated (HR and 95% CI > 1) with the outcome but failed to meet the low to moderate heterogeneity (*I^2^*) criteria.

Considering the small number of studies that were pooled, publication bias was not evaluated.

## 4. Discussion

To the best of our knowledge, this study is the first to use a quantitative approach that combines individual blood metabolites to evaluate their prognostic value in patients with HF.

McGranaghan et al. performed a meta-analysis of metabolomic features associated with incident cardiovascular disease (CVD) in patients without CVD or patients with risk factors [36]. The authors used random-effects models to combine measures gathered per metabolite family (glycerolipids, glycerophospholipids, sphingolipids, acylcarnitines, amino acids, cholesterol esters, and fatty acids), metabolite scores, and by combining all metabolites in a single overall HR, thus losing the specific information of individual metabolites. Ruiz-Canela et al. conducted a systematic review of metabolomic features and incident CVD (MI, stroke, and/or CV death) in patients with and without CVD [37]. The authors found that acylcarnitines, dicarboxylacyl-carnitines, and several amino acid and lipid classes were associated with CVD risk, although the addition of such biomarkers resulted in a modest improvement in CVD prediction beyond traditional risk factors.

A brief context of metabolites found to be relevant in this work is discussed in the following subsections.

### 4.1. Symmetric Dimethylarginine (SDMA) and SDMA/Arginine

Symmetric Dimethylarginine is a derivative of L-arginine generated by the post-translational methylation of arginine residues, such as its isomer, Asymmetric Dimethylarginine (ADMA). This methylation occurs by the action of enzymes from the protein arginine methyltransferases family [38]. Both free ADMA and SDMA are released following proteolysis, although arising from different metabolic pathways, involving protein arginine methyltransferase type 1 and 2 (PRMT1, PRMT2), respectively [39].

It has been reported that ADMA, and to a much lower extent SMDA, diminish nitric oxide (NO) bioavailability. ADMA directly inhibits the NO synthase activity [39], and SDMA may interfere with the use of the enzyme substrate L-arginine, which leads to an indirect inhibition [40]. NO plays a vital role in cardiovascular physiology, linked to endothelial function, cardiac contractibility, and cardiac protection [41]. In the context of HF, NO inhibits the chronic β-adrenergic response of ventricular myocardium, which is enhanced in this condition [41]. ADMA and SDMA have been previously shown to be independent markers of all-cause mortality across different types of populations, including those with CVD [42]. Bode-Bo et al. established a relationship between high SDMA plasma levels and patients with coronary artery disease [40], while Potočnjak et al. linked high SDMA levels to mortality in acute HF patients [43].

### 4.2. Putrescine

**Figure 3:**
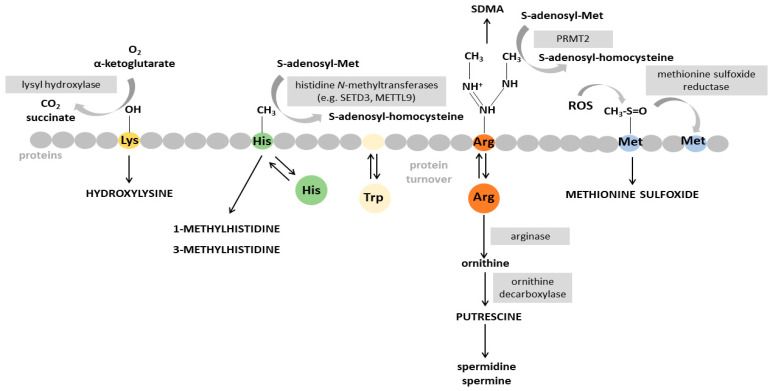
Metabolic pathways and reactions. Arg: Arginine; His: Histidine; Lys: Lysine; Met: Methionine; ROS: Reactive oxygen species; Trp: Tryptophan.

Putrescine is produced by ornithine decarboxylation, which acts on a metabolite produced in the breakdown of arginine (Figure 3). This polyamine serves as one of the precursors for other polyamines, including spermidine and spermine [44]. Polyamines are strongly positively charged at physiological pH and bind to acidic sites on cellular macromolecules including proteins, nucleic acids, and phospholipid membranes, regulating their activity [45]. Therefore, polyamines regulate several biological processes, such as cell division, apoptosis, and gene transcription processes [46], and have been implicated in cardiac hypertrophy in animal models [47]. In humans, a study involving 17 heart failure patients demonstrated an association between the enzymatic activity of ornithine decarboxylation and left atrial hemodynamic overload, along with increased levels of polyamines and improvement in ventricular inotropism [48]. These findings suggest that polyamine production and subsequent putrescine represent early events in cardiac hypertrophy. Cardiac hypertrophy is an adaptive response to increased functional demand on the heart and may be the result of a large variety of stimuli [49]. Diseases such as hypertension and myocardial infarction lead to pathological cardiac hypertrophy which can ultimately induce HF [49]. Given the implications of polyamines, including putrescine, in cardiac hypertrophy, they hold potential as markers for hypertrophy progression and, ultimately, HF.

### 4.3. N-Methyl-Histidine

Protein methylation primarily targets basic amino acid residues like arginine, lysine, and histidine [50]. Histidine methylation extends beyond the histone code. This post-translational modification has been identified by mass spectrometry in non-histone proteins such as actin and myosin [50]. The histidine N-methyltransferase SETD3 targets His73 of actin, methylating this amino acid residue at N3 of the imidazole ring, which has an impact on muscle contractibility [50]. METTL9 catalyzes the methylation of histidine at N1, forming 1-methylhistidine, in the inflammatory protein S100A9 (Figure 3) [51]. Notably, elevated levels of free 1-methylhistidine have already been linked to hypertension [52], diastolic dysfunction [53], and heart failure [54]. These associations may imply changes in the turnover rates of proteins methylated on histidine residues.

### 4.4. Hydroxylysine

Hydroxylysine, a hydroxylated derivative of lysine, is present in various types of collagens [55]. This hydroxylation is catalyzed by lysyl hydroxylases and is critical for the following glycosylation and in determining the fate of covalent cross-linking, which contributes to the stiffness and resiliency of collagens, thereby influencing their structural properties (Figure 3) [55]. Free forms of hydroxylysine can arise through proteolytic degradation of collagen, and the urinary excretion of 5-Hydroxylysine serves as an index of collagen degradation. Elevated levels of urinary hydroxylysine are indicative of more rapid or extensive collagen degradation [56]. Cardiac collagen remodeling is a crucial step in HF progression, with higher blood levels of its degradation markers being observed in HF populations [57]. Although hydroxylysine is not an exclusive component of heart-derived collagen, it is a key component and a possible marker of collagen synthesis and turnover, especially in states associated with increased myocardial fibrosis, such as HF, suggesting altered collagen pathway degradation in patients with worse prognosis.

### 4.5. Methionine Sulfoxide

Methionine sulfoxide arises through the oxidation of methionine residues in proteins by reactive oxygen species (ROS) under various physiological or pathological conditions [58]. Its levels are dependent on the redox status in the organ, and on the methionine sulfoxide reductase system that can reduce methionine sulfoxide to methionine (Figure 3). The role of free methionine sulfoxide in the regulation of cellular processes is poorly comprehended [58]. Nevertheless, because of its connection to ROS, methionine sulfoxide is recognized as a biomarker of oxidative stress in various conditions connected to oxidative stress, for example, aging, type 2 diabetes, chronic renal failure, and ischemic conditions [59,60]. In a cross-sectional analysis of the Bogalusa Heart Study, a population-based study that analyzed the natural course of CVD across the lifespan, methionine sulfoxide was associated with the presence of diastolic dysfunction [53]. The study suggested that methionine sulfoxide could be released from cardiac myocyte protein turnover in the presence of left ventricular diastolic dysfunction or systemic endothelial dysfunction. Furthermore, reduced levels of methionine sulfoxide reductase have been observed during ischemia, possibly implicating this enzyme in vascular disease and cardiac ischemia [60]. In our meta-analysis, methionine sulfoxide was associated with poor outcomes, although its role in HF pathology remains unclear.

### 4.6. Histidine

Histidine is an essential amino acid used in the biosynthesis of proteins [61]. It contains an imidazole functional group capable of scavenging ROS generated during acute inflammatory response [62]. This property imparts histidine with antioxidant and anti-inflammatory characteristics [63]. Liu et al., showed that histidine levels were reduced in HF patients when compared with healthy controls, demonstrating its sensitivity in distinguishing the two groups [64]. Anguita et al. demonstrated that plasma levels of three metabolites, including formate, lactate, and histidine, were determinant for the classification between decompensated or stable heart failure with reduced ejection fraction (HFpEF) [65]. Low histidine levels are associated with poor outcomes, possibly implicating a disruption in inflammatory processes in patients with HF.

### 4.7. Tryptophan

Tryptophan is an essential amino acid utilized in protein biosynthesis and is mainly catabolized in pro-inflammatory states [66], generating kynurenine and hydroxykynurenine, among others [67]. In previous works, higher levels of tryptophan were found to be associated with lower HF risk, while kynurenine and hydroxykynurenine, showed the opposite trend, plausibly linked to the tryptophan–kynurenine pathway and the inflammatory state in HF patients [68]. Inflammation converts tryptophan into kynurenine and hydroxykynurenine, resulting in reduced levels of tryptophan and increased levels of kynurenine and hydroxykynurenine [67]. These two tryptophan catabolites were already linked to HF prognosis [28,34,69], but these findings were not included in our meta-analysis as the studies did not match all the inclusion criteria (lack of the pre-specified data preprocessing).

### 4.8. Taking Metabolomics to a Clinical Setting

Metabolomics is one of the youngest “omics” fields to emerge and is considered a promising tool in a clinical scenario [12], as it offers the key advantage of simultaneous measurement of hundreds of metabolites in a single experimental run. As these molecules participate in different biological processes, their change may pinpoint perturbations in specific pathways, making metabolomics valuable for hypothesis-generation studies. Moreover, personalized metabolomics further recognizes the importance of each individual’s traits and characteristics, such as comorbidities (e.g., obesity, hypertension, diabetes) and lifestyle (e.g., dietary and exercise habits) in defining their clinical trajectory and outcome. Thus, metabolomics may play an important role in the understanding of the pathophysiology and mechanisms of complex multifactorial diseases, shedding light on new pharmaceutical research towards the identification of novel therapeutic agents.

However, despite such huge data sets collected to date and such promising perspectives of personalized metabolomics, there are yet no clinical metabolomics applications [70]. One of the main problems discussed in the literature and corroborated in this study is the lack of standardization across all stages of research. Only through the standardization of analytical strategies, data statistical analysis and reporting, findings may be compared and combined [70]. The Metabolomics Standards Initiative (MSI) [71] and the Framework Programme 7 EU Initiative ‘coordination of standards in metabolomics’ (COSMOS) [72] have already pinpointed the importance of standardization in all stages of the metabolomic framework. The metabolomic community would greatly benefit from having standard protocols, including all analytical steps (sample collection, handling, and analysis) and data handling (pre-processing and statistical modeling). Not less important is the need for data sharing standards, for instance, through the use of checklists for data and metadata [71] that ensure compliance with FAIR principles, to envisage adequate exchange, comparison and re-utilization of metabolomics datasets [72]. Another significant drawback, although with few exceptions in the literature, is the lack of reporting standards, with most metabolomics studies presenting their findings in non-quantitative scales (i.e., log-transformed, standardized, or normalized to total intensities) instead of quantitative ones (mg/dL or mmol/dL), impacting the potential clinical applications of such findings.

### 4.9. Study Limitations

The present review and meta-analysis are subject to several limitations, including (1) the lack of studies with comparable and similar data treatment, thereby restricting the range of metabolites that could be included; (2) the existence of heterogeneity in terms of the study population and endpoints; and (3) the absence of a subgroup or sensitivity analysis for the metabolites that were subjected to meta-analysis.

## 5. Conclusions

We conducted a comprehensive review of blood metabolites in patients with HF and combined their prognostic value through meta-analysis. We identified seven individual metabolites and one metabolite ratio that were significantly associated with the prognosis of patients with HF. Metabolites such as histidine and tryptophan emerged as protective factors, while others were associated with poorer outcomes. However, there are significant challenges towards the implementation of (personalized) metabolomics in clinical practice. As the first comprehensive study to summarize individual blood metabolites and to quantitatively assess their prognostic value in HF, our work emphasizes the importance of further research on metabolites and metabolomics in cardiovascular disease research.

## Figures and Tables

**Table 1:** Description of the population included in each study.

Study Design and Name	Population	Primary Outcome	No. of Events/ No. of Patients	Median Follow-Up (Years)	Platform	Sample	Ref.
Cohort	Patients > 18 years with first-time STEMI, presented within 12 h of onset of chest pain, and AHF. Exclusion criteria were as follows: renal or liver dysfunction; use of amino acid-enriched alimentary integrators in the preceding 3 months; less than 30-day follow-up.	All-cause mortality or heart failure hospitalization	53/138	2.04	LC-MS	Plasma	[31]
GRADE cohort	Patients > 18 years with the following: ischemic or non-ischemic cardiomyopathy; significant left ventricular systolic dysfunction; increased left ventricular size; implanted ICD for primary prevention; serum available for metabolic profiling.	All-cause mortality	39/240	3.7	UPLC-MS/MS	serum	[32]
PROSE-ICD cohort	Patients 18–80 years of age referred for primary prevention ICD implantation who met one of the following criteria: MI > 40 days prior to implant with an EF < 30% and stable NYHA Class I–III HF; ischemic or nonischemic cardiomyopathy with an EF < 35% and NYHA Class II or III heart failure; EF < 35% with NYHA Class II–IV HF undergoing guideline-indicated implantation of a cardiac resynchronization therapy device with an ICD; serum available for metabolic profiling.	All-cause mortality	120/402	5.5	UPLC-MS/MS	serum	[32]
PROSE-ICD cohort	Patients 18–80 years of age referred for primary prevention ICD implantation who met one of the following criteria: MI > 40 days prior to implant with an EF < 30% and stable NYHA Class I–III HF; ischemic or nonischemic cardiomyopathy with an EF < 35% and NYHA Class II or III HF; EF < 35% with NYHA Class II–IV HF undergoing guideline-indicated implantation of a cardiac resynchronization therapy device with an ICD; serum available for metabolic profiling.	All-cause mortality	161/479	6.3	LC-MS	serum	[33]
Retrospective Cohort	HF patients diagnosed according to the 2016 ESC Guidelines. Exclusion criteria were as follows: pulmonary artery hypertension; acute myocarditis; CKD at stage 5; missing data for amino acid profiling; follow-up period < 30 days.	All-cause death orunscheduled readmission due to worsening HF or lethal arrhythmia	40/301	1.04 *	UPLC	Plasma	[34]

*: mean follow-up time. AHF: acute heart failure; CKD: chronic kidney disease; EF: ejection fraction; ICD: implantable cardioverter-defibrillator; LC-MS: liquid chromatography-tandem mass spectrometry; MI: myocardial infarction; NYHA: New York Heart Association; UPLC: ultra-performance liquid chromatography; UPLC-MS/MS: ultra-performance liquid chromatography-tandem mass spectrometry; STEMI: ST-elevation myocardial infarction.

## Data Availability

All data used in this article are available in the Supplementary Materials.

## Supplementary Materials

The following supporting information can be downloaded at: https://www.mdpi.com/article/10.3390/ijms25115693/s1.
