# Supplementary material for: Metabolomics and Cardiovascular Risk in Patients with Heart Failure: A Systematic Review and Meta-Analysis
Source: Int J Mol Sci. 2024 May 23;25(11):5693. doi: 10.3390/ijms25115693 (PMC11172189; doi:10.3390/ijms25115693)
Supplement: Supplementary file 1 [file ijms-25-05693-s001.zip › ijms-2982413-supplementary.pdf]

Table S1. Population characteristics of each included study.

Cohort name, year and reference	Du et al., 2018, [31]			PROSE-ICD cohort, 2016 [32]				GRADE cohort, 2016 [32]				PROSE-ICD cohort, 2016 [33]				Kouzu et al., 2021 [34]			
	Event +	Event -	p*	Total	Event -	Event +	p	Total	Event -	Event +	p	total	Event -	Event +	p	All	Event +	Event -	p
N	85	53		402	282	120		240	201	39		479	318	161		301	40	261	
Age (year)	61.4 ± 10.0	63.1 ± 8.8	0.300	60.1±12.8	57.8±11.9	65.5±13.2	<0.001	62.5±11.8	61.6±11.3	67.3±13.2	0.006	60.1 ± 12.8	57.1 ± 12.2	66.1 ± 12.0	<0.001	70.2 (14.5)	72.2 (15.7)	69.9 (14.3)	0.342
Sex (Male)	52 (61.2)	26 (49.1)	0.004	296 (73.6)	196 (69.5)	100 (83.3)	0.004	185 (77.1)	152 (75.6)	33 (84.6)	0.22	349 (72.9)	216 (67.9)	133 (82.6)	0.001	177 (59)	23 (58)	154 (59)	0.857
Body mass index (kg/m^2)	25.6 ± 2.0	26.0 ± 2.0	0.276	29.4±6.5	29.7±6.7	28.5±6.0	0.08	28.7±5.5	29.1±5.3	26.5±6.3	0.009	NR	NR	NR	NR	23.2 (4.1)	22.1 (4.2)	23.3 (4.1)	0.087
Ejection fraction (%)	45.0 ± 5.0	43.9 ± 6.2	0.259	21.6±7.5	21.9±7.6	20.6±7.1	0.11	20.4±6.6	20.5±6.5	19.6±7.0	0.47	21.9 ± 7.5	22.1 ± 7.7	21.6 ± 7.2	0.51	46.6 (16.0)	42.9 (17.3)	47.2 (15.7)	0.120
NHYA class																			
Class I	NR	NR	NR	59 (14.7)	48 (17.0)	11 (9.2)	0.004	45 (18.8)	39 (19.4)	6 (15.4)	0.51	70 (14.6)	57 (17.9)	13 (8.1)	0.01	NR	NR	NR	NR
Class II	NR	NR	NR	162 (40.3)	123 (43.6)	39 (32.5)		125 (52.1)	107 (53.2)	18 (46.2)		192 (40.1)	132 (41.5)	60 (37.3)		NR	NR	NR	NR
Class III	NR	NR	NR	180 (44.8)	110 (39.0)	70 (58.3)		69 (28.8)	54 (26.9)	15 (38.5)		216 (45.1)	128 (40.3)	88 (54.7)		84 (28)	17 (43)	67 (26)	0.027
Class IV	NR	NR	NR	1 (0.2)	1 (0.4)	0 (0.0)		0 (0.0)	0 (0.0)	0 (0.0)		1 (0.2)	1 (0.3)	0 (0.0)					
Ischaemic cardiomyopathy	NR	NR	NR	216 (53.7)	142 (50.4)	74 (61.7)	0.04	168 (70.0)	138 (68.7)	30 (76.9)	0.30	262 (54.7)	151 (47.5)	111 (68.9)	<0.001	43 (14)	7 (18)	36 (14)	0.873
Atrial fibrillation	NR	NR	NR	103 (25.6)	59 (20.9)	44 (36.7)	0.001	38 (15.8)	29 (14.4)	9 (23.1)	0.16	119 (24.8)	59 (18.6)	60 (37.3)	<0.001	114 (38)	18 (45)	96 (37)	0.318
Diabetes	17 (20.0)	15 (26.4)	0.004	128 (31.8)	82 (29.1)	46 (38.3)	0.07	74 (30.8)	62 (30.8)	12 (30.8)	0.91	154 (32.2)	82 (25.8)	72 (44.7)	<0.001	107 (36)	16 (40)	91 (35)	0.528

Table S1. *continued*

Cohort name, year and reference	Du et al., 2018, [31]			PROSE-ICD cohort, 2016 [32]				GRADE cohort, 2016 [32]				PROSE-ICD cohort, 2016 [33]				Kouzu et al., 2021 [34]			
	Event +	Event -	p*	Total	Event -	Event +	p	Total	Event -	Event +	p	total	Event -	Event +	p	All	Event +	Event -	p
Hypertension	38 (44.7)	25 (47.2)	0.780	242 (60.2)	165 (58.5)	77 (64.2)	0.29	160 (66.7)	135 (67.2)	25 (64.1)	0.83	289 (60.3)	181 (56.9)	108 (67.1)	0.03	185 (61)	23 (58)	162 (62)	0.580
Chronic kidney disease	NR	NR	NR	111 (27.6)	55 (19.5)	56 (46.7	<0.001	NR	NR	NR	NR	136 (28.4)	63 (19.8)	73 (45.3)	<0.001	174 (58)	35 (88)	139 (53)	<0.001
Medications																			
Aspirin	NR	NR	NR	264 (65.7)	185 (65.6)	79 (65.8)	0.96	NR	NR	NR	NR	318 (66.4)	205 (64.5)	113 (70.2)	0.21	NR	NR	NR	NR
ACE-I/ARB	70 (82.4)	45 (84.9)	0.696	291 (72.4)	203 (72.0)	88 (73.3)	0.78	184 (76.7)	154 (76.6)	30 (76.9)	0.97	349 (72.9)	226 (71.1)	123 (76.4)	0.22	174 (58)	17 (43)	157 (60)	0.035
Beta-blocker	72 (84.7)	43 (81.1)	0.584	357 (88.8)	254 (90.1)	103 (85.8)	0.22	213 (88.8)	180 (89.6)	33 (84.6)	0.45	429 (89.6)	288 (90.6)	141 (87.6)	0.31	218 (72)	29 (73)	189 (72)	0.991
Thiazide/loop diuretics	NR	NR	NR	275 (68.4)	188 (66.7)	87 (72.5)	0.25	164 (68.3)	133 (66.2)	31 (79.5)	0.19	333 (69.5)	214 (67.3)	119 (73.9)	0.14	NR	NR	NR	NR
Lopp diuretics	NR	NR	NR	NR	NR	NR	NR	NR	NR	NR	NR	NR	NR	NR	NR	163 (54)	29 (73)	134 (51)	0.012
Aldosterone antagonist	69 (81.2)	41 (77.4)	0.792	99 (24.6)	67 (23.8)	32 (26.7)	0.54	64 (26.7)	57 (28.4)	7 (17.9)	0.32	122 (25.5)	77 (24.2)	45 (28.0)	0.38	135 (45)	26 (65)	109 (42)	0.006
Laboratory data																			
NT-proBNP (pg/mL)	3722.9 ± 2149.5	2616.8 ± 2993.5	<0.001	NR	NR	NR	NR	NR	NR	NR	NR	NR	NR	NR	NR	995 (467–2150)	1387 (840–4227)	940 (416–2023	0.016

Data are presented as mean ± standard deviation or median (IQR) and are expressed as frequency and percentage. p* p value from logistic regression analysis; ACE-I/ARB: Angiotensin-converting-enzyme inhibitors/ Angiotensin II receptor blocker; LVEF: Left ventricle ejection fraction; NYHA: New York Heart Association. NR: Not reported.

Table S2. Metabolite data from the included studies.

Variables used for adjustment	Metabolites	HR	CI95%	Class	Subclass	Included in meta-analysis	Ref.	HMDB ID
c	11,12-DiHETrE	1.12	[0.95;1.31]	Fatty Acyls	Eicosanoids	No	[33]	HMDB0002314
c	11.12-DIHETrE/FA(20:4-w6)	1.11	[0.95;1.30]	NA	NA	No	[33]	NA
c	11-HETE	1.00	[0.82;1.22]	Fatty Acyls	Eicosanoids	No	[33]	HMDB0004682
c	11-HETE/FA(20:4-w6)	1.00	[0.82;1.21]	NA	NA	No	[33]	NA
c	12,13-DiHOME	0.94	[0.80;1.11]	Fatty Acyls	Fatty acids and conjugates	No	[33]	HMDB0004705
c	12,13-DIHOME/12,13-EpOME	0.96	[0.80;1.14]	NA	NA	No	[33]	NA
c	12,13-DIHOME/FA(18:2)	0.98	[0.84;1.15]	NA	NA	No	[33]	NA
c	12,13-EpOME	0.98	[0.82;1.15]	Fatty Acyls	Fatty acids and conjugates	No	[33]	HMDB0004702
c	12,13-EpOME/FA(18:2)	1.01	[0.86;1.19]	NA	NA	No	[33]	NA
c	125-HEPE/FA(20:5-w3)	1.07	[0.91;1.26]	NA	NA	No	[33]	NA
c	12S-HHTrE	0.85	[0.71;1.02]	Fatty Acyls	Eicosanoids	No	[33]	HMDB0012535

c	12-HETE	1.05	[0.89;1.24]	NA	NA	No	[33]	HMDB0006111
c	12-HETE/FA(20:4-W6)	1.05	[0.89;1.23]	Fatty Acyls	Eicosanoids	No	[33]	NA
c	12S-HEPE	0.96	[0.81;1.14]	Fatty Acyls	Fatty acids and conjugates	No	[33]	HMDB0244454
c	13,14-dihydro-PGF2a	0.90	[0.76;1.06]	Fatty Acyls	Eicosanoids	No	[33]	HMDB0004239
c	13-HODE	1.03	[0.84;1.27]	Fatty Acyls	Lineolic acids and derivatives	No	[33]	HMDB0004667
c	13-HODE/FA(18:2)	1.08	[0.89;1.30]	NA	NA	No	[33]	NA
c	14,15-DIHETrE	1.10	[0.93;1.31]	Fatty Acyls	Eicosanoids	No	[33]	HMDB0004667
c	14.15-DIHETrE/FA(20:4-w6)	1.08	[0.92;1.28]	NA	NA	No	[33]	NA
c	15-HETE	1.00	[0.81;1.22]	Fatty Acyls	Eicosanoids	No	[33]	HMDB0003876
c	15-HETE/FA(20:4-w6)	0.99	[0.81;1.20]	NA	NA	No	[33]	NA
c	15S-HEPE	1.14	[0.94;1.38]	Fatty Acyls	Eicosanoids	No	[33]	HMDB0062296
c	15S-HEPE/FA(20:5-w3)	1.28	[1.06;1.55]	NA	NA	No	[33]	NA
c	15S-HETrE	1.13	[0.95;1.34]	Fatty Acyls	Eicosanoids	No	[33]	HMDB0005045

c	15S-HETrE/FA(20:3-w3w6)	1.14	[0.96;1.34]	NA	NA	No	[33]	NA
c	17,18-DIHETE	1.06	[0.91;1.23]	Fatty Acyls	Eicosanoids	No	[33]	HMDB0010211
c	17,18-DIHETE/FA(20:5-w3)	1.24	[1.05;1.46]	NA	NA	No	[33]	NA
c	19,20-DiHDPA	1.10	[0.93;1.30]	Fatty Acyls	Fatty acids and conjugates	No	[33]	HMDB0010214
c	19.20-DIHDPA/FA(20:5-w3)	1.27	[1.06;1.51]	NA	NA	No	[33]	NA
c	20-HETE	1.10	[0.92;1.32]	Fatty Acyls	Fatty acids and conjugates	No	[33]	HMDB0005998
c	20-HETE/FA(20:4-w6)	1.11	[0.94;1.31]	NA	NA	No	[33]	NA
c	20-hydroxy-LTB4	0.85	[0.70;1.05]	Fatty Acyls	Eicosanoids	No	[33]	HMDB0001509
c	20-hydroxy-LTB4/FA(20:4-w6)	0.85	[0.70;1.04]	NA	NA	No	[33]	NA
d	2-aminoadipic acid	1.06	[0.68;1.65]	Carboxylic acids and derivatives	Amino acids, peptides, and analogues	Yes	[32]	HMDB0302754
e	2-aminoadipic acid	0.87	[0.70;1.09]	Carboxylic acids and derivatives	Amino acids, peptides, and analogues	Yes	[32]	HMDB0302754

a	3-methyl histidine	2.89	[1.82;4.50]	Carboxylic acids and derivatives	Amino acids, peptides, and analogues	Yes	[34]	HMDB0000479
d	3-methyl histidine	1.65	[1.09;2.51]	Carboxylic acids and derivatives	Amino acids, peptides, and analogues	Yes	[32]	HMDB0000479
e	3-methyl histidine	1.06	[0.88;1.27]	Carboxylic acids and derivatives	Amino acids, peptides, and analogues	Yes	[32]	HMDB0000479
c	5,6-DiHETrE	1.14	[0.97;1.35]	Fatty Acyls	Eicosanoids	No	[33]	HMDB0002343
c	5,6-DIHETrE/FA(20:4-w6)	1.13	[0.95;1.33]	NA	NA	No	[33]	NA
c	5-HETE	1.6	[0.89;1.26]	Fatty Acyls	Eicosanoids	No	[33]	HMDB0011134
c	5-HETE/FA(20:4-w6)	1.05	[0.88;1.26]	NA	NA	No	[33]	NA
c	5S-HEPE	1.01	[0.86;1.19]	Fatty Acyls	Eicosanoids	No	[33]	HMDB0062408
c	5S-HEPE/FA(20:5-w3)	1.21	[1.02;1.44]	NA	NA	No	[33]	NA
c	6-trans-LTB4	0.98	[0.80;1.19]	Fatty Acyls	Eicosanoids	No	[33]	HMDB0005087
c	6-trans-LTB4/FA(20:4-w6)	0.98	[0.80;1.19]	NA	NA	No	[33]	NA
c	8,9-DiHETrE	1.10	[0.94;1.29]	Fatty Acyls	Eicosanoids	No	[33]	HMDB0002311

c	8,9-DIHETrE/FA(20:4-W6)	1.09	[0.93;1.28]	NA	NA	No	[33]	NA
c	8-HETE	1.02	[0.85;1.23]	Fatty Acyls	Eicosanoids	No	[33]	HMDB0004679
c	8-HETE/FA(20:4-w6)	1.02	[0.85;1.22]	NA	NA	No	[33]	NA
c	9,10,13-TriHOME	1.03	[0.86;1.24]	Fatty Acyls	Fatty acids and conjugates	No	[33]	HMDB0004710
c	9,10,13-TriHOME/FA(18:2)	1.06	[0.89;1.26]	NA	NA	No	[33]	NA
c	9,10-DiHOME	0.87	[0.72;1.04]	Fatty Acyls	Fatty acids and conjugates	No	[33]	HMDB0004704
c	9,10-DiHOME/9,10-EpOME	0.85	[0.71;1.03]	NA	NA	No	[33]	NA
c	9,10-DIHOME/FA(18:2)	0.91	[0.77;1.08]	NA	NA	No	[33]	NA
c	9,10-EpOME	1.01	[0.85;1.20]	Fatty Acyls	Fatty acids and conjugates	No	[33]	HMDB0004701
c	9,10-EpOME/FA(18:2)	1.05	[0.89;1.25]	NA	NA	No	[33]	NA
c	9,12,13-TriHOME	1.06	[0.88;1.27]	Fatty Acyls	Fatty acids and conjugates	No	[33]	HMDB0004708
c	9,12,13-TriHOME/FA(18:2)	1.08	[0.91;1.29]	NA	NA	No	[33]	NA

c	9-HODE	1.04	[0.86;1.25]	Fatty Acyls	Lineolic acids and derivatives	No	[33]	HMDB0062652
c	9-HODE/FA(18:2)	1.08	[0.90;1.29]	NA	NA	No	[33]	NA
c	9-HOTrE	0.99	[0.81;1.21]	Fatty Acyls	Lineolic acids and derivatives	No	[33]	HMDB0247705
c	9-HOTrE/FA(18:3-w3w6)	1.07	[0.89;1.29]	NA	NA	No	[33]	NA
d	ADMA	1.24	[0.73;2.11]	Carboxylic acids and derivatives	Amino acids, peptides, and analogues	Yes	[32]	HMDB0001539
e	ADMA	1.16	[0.83;1.62]	Carboxylic acids and derivatives	Amino acids, peptides, and analogues	Yes	[32]	HMDB0001539
d	ADMA/arginine	1.16	[0.74;1.81]	NA	NA	Yes	[32]	NA
e	ADMA/arginine	1.07	[0.87;1.30]	NA	NA	Yes	[32]	NA
b	Alanine	1.11	[0.84;1.47]	Carboxylic acids and derivatives	Amino acids, peptides, and analogues	Yes	[31]	HMDB0000161
d	Alanine	0.86	[0.58;1.28]	Carboxylic acids and derivatives	Amino acids, peptides, and analogues	Yes	[32]	HMDB0000161

e	Alanine	0.75	[0.60;0.94]	Carboxylic acids and derivatives	Amino acids, peptides, and analogues	Yes	[32]	HMDB0000161
b	Arginine	0.87	[0.67;1.12]	Carboxylic acids and derivatives	Amino acids, peptides, and analogues	Yes	[31]	HMDB0000517
d	Arginine	0.93	[0.59;1.48]	Carboxylic acids and derivatives	Amino acids, peptides, and analogues	Yes	[32]	HMDB0000517
e	Arginine	1.03	[0.79;1.35]	Carboxylic acids and derivatives	Amino acids, peptides, and analogues	Yes	[32]	HMDB0000517
b	Asparagine	1.15	[0.85;1.54]	Carboxylic acids and derivatives	Amino acids, peptides, and analogues	Yes	[31]	HMDB0000168
d	Asparagine	0.98	[0.62;1.55]	Carboxylic acids and derivatives	Amino acids, peptides, and analogues	Yes	[32]	HMDB0000168
e	Asparagine	0.99	[0.76;1.28]	Carboxylic acids and derivatives	Amino acids, peptides, and analogues	Yes	[32]	HMDB0000168

d	Aspartic acid	0.90	[0.56;1.47]	Carboxylic acids and derivatives	Amino acids, peptides, and analogues	Yes	[32]	HMDB0000191
e	Aspartic acid	1.00	[0.83;1.21]	Carboxylic acids and derivatives	Amino acids, peptides, and analogues	Yes	[32]	HMDB0000191
b	BCAA	2.67	[1.86;3.83]	Carboxylic acids and derivatives	Amino acids, peptides, and analogues	No	[31]	NA
b	Citrulline	1.29	[0.94;1.78]	Carboxylic acids and derivatives	Amino acids, peptides, and analogues	Yes	[31]	HMDB0000904
d	Citrulline	1.67	[0.97;2.98]	Carboxylic acids and derivatives	Amino acids, peptides, and analogues	Yes	[32]	HMDB0000904
e	Citrulline	0.94	[0.77;1.16]	Carboxylic acids and derivatives	Amino acids, peptides, and analogues	Yes	[32]	HMDB0000904
b	Creatine	1.09	[0.80;1.50]	Carboxylic acids and derivatives	Amino acids, peptides, and analogues	No	[31]	HMDB0000064

d	DL-5-hydroxylysine	1.24	[0.82;1.89]	Carboxylic acids and derivatives	Amino acids, peptides, and analogues	Yes	[32]	HMDB0000450
e	DL-5-hydroxylysine	1.25	[1.04;1.51]	Carboxylic acids and derivatives	Amino acids, peptides, and analogues	Yes	[32]	HMDB0000450
d	Ethanolamine	0.91	[0.60;1.40]	Organonitrogen compounds	Amines	Yes	[32]	HMDB0000149
e	Ethanolamine	1.14	[0.87;1.50]	Organonitrogen compounds	Amines	Yes	[32]	HMDB0000149
b	Glutamate	1.28	[0.97;1.67]	Carboxylic acids and derivatives	Amino acids, peptides, and analogues	Yes	[31]	HMDB0060475
d	Glutamate	1.00	[0.60;1.67]	Carboxylic acids and derivatives	Amino acids, peptides, and analogues	Yes	[32]	HMDB0060475
e	Glutamate	1.09	[0.87;1.37]	Carboxylic acids and derivatives	Amino acids, peptides, and analogues	Yes	[32]	HMDB0060475

b	Glutamine	0.98	[0.81;1.20]	Carboxylic acids and derivatives	Amino acids, peptides, and analogues	Yes	[31]	HMDB0000641
d	Glutamine	0.79	[0.54;1.14]	Carboxylic acids and derivatives	Amino acids, peptides, and analogues	Yes	[32]	HMDB0000641
e	Glutamine	0.70	[0.48;1.02]	Carboxylic acids and derivatives	Amino acids, peptides, and analogues	Yes	[32]	HMDB0000641
b	Glycine	1.19	[0.91;1.55]	Carboxylic acids and derivatives	Amino acids, peptides, and analogues	Yes	[31]	HMDB0000123
d	Glycine	0.97	[0.61;1.55]	Carboxylic acids and derivatives	Amino acids, peptides, and analogues	Yes	[32]	HMDB0000123
e	Glycine	1.03	[0.78;1.37]	Carboxylic acids and derivatives	Amino acids, peptides, and analogues	Yes	[32]	HMDB0000123
d	Glycylglycine	0.98	[0.65;1.48]	Carboxylic acids and derivatives	Amino acids, peptides, and analogues	Yes	[32]	HMDB0011733

e	Glycylglycine	1.15	[0.96;1.37]	Carboxylic acids and derivatives	Amino acids, peptides, and analogues	Yes	[32]	HMDB0011733
b	Histidine	0.86	[0.66;1.13]	Carboxylic acids and derivatives	Amino acids, peptides, and analogues	Yes	[31]	HMDB0000177
d	Histidine	0.74	[0.48;1.16]	Carboxylic acids and derivatives	Amino acids, peptides, and analogues	Yes	[32]	HMDB0000177
e	Histidine	0.70	[0.60;0.83]	Carboxylic acids and derivatives	Amino acids, peptides, and analogues	Yes	[32]	HMDB0000177
d	Homoserine	1.24	[0.75;2.04]	Carboxylic acids and derivatives	Amino acids, peptides, and analogues	Yes	[32]	HMDB0000719
e	Homoserine	0.92	[0.75;1.12]	Carboxylic acids and derivatives	Amino acids, peptides, and analogues	Yes	[32]	HMDB0000719
b	Hydroxyproline	1.19	[0.93;1.52]	Carboxylic acids and derivatives	Amino acids, peptides, and analogues	Yes	[31]	HMDB0000725

d	Hydroxyproline	1.52	[1.08;2.14]	Carboxylic acids and derivatives	Amino acids, peptides, and analogues	Yes	[32]	HMDB0000725
e	Hydroxyproline	0.98	[0.81;1.19]	Carboxylic acids and derivatives	Amino acids, peptides, and analogues	Yes	[32]	HMDB0000725
b	Isoleucine	2.72	[1.90;3.90]	Carboxylic acids and derivatives	Amino acids, peptides, and analogues	Yes	[31]	HMDB0000172
d	Isoleucine	0.91	[0.60;1.39]	Carboxylic acids and derivatives	Amino acids, peptides, and analogues	Yes	[32]	HMDB0000172
e	Isoleucine	0.95	[0.77;1.16]	Carboxylic acids and derivatives	Amino acids, peptides, and analogues	Yes	[32]	HMDB0000172
b	Kynurenine	1.18	[0.89;1.55]	Organooxygen compounds	Carbonyl compounds	Yes	[31]	HMDB0000684
d	Kynurenine	1.73	[1.32;2.27]	Organooxygen compounds	Carbonyl compounds	Yes	[32]	HMDB0000684
e	Kynurenine	1.31	[1.06;1.63]	Organooxygen compounds	Carbonyl compounds	Yes	[32]	HMDB0000684

d	Kynurenine/tryptophan	2.01	[1.48;2.73]	NA	NA	Yes	[32]	NA
e	Kynurenine/tryptophan	1.43	[1.18;1.72]	NA	NA	Yes	[32]	NA
b	Leucine	2.75	[1.81;4.19]	Carboxylic acids and derivatives	Amino acids, peptides, and analogues	Yes	[31]	HMDB0000687
d	Leucine	0.72	[0.45;1.14]	Carboxylic acids and derivatives	Amino acids, peptides, and analogues	Yes	[32]	HMDB0000687
e	Leucine	0.77	[0.62;0.96]	Carboxylic acids and derivatives	Amino acids, peptides, and analogues	Yes	[32]	HMDB0000687
c	LTB4	0.88	[0.72;1.08]	Fatty Acyls	Eicosanoids	No	[33]	HMDB0001085
c	LTB4/FA(20:4-w6)	0.88	[0.72;1.08]	NA	NA	No	[33]	NA
c	LTE4	0.95	[0.80;1.13]	Fatty Acyls	Eicosanoids	No	[33]	HMDB0002200
c	LTE4/FA(20:4-w6)	0.95	[0.80;1.13]	NA	NA	No	[33]	NA
b	Lysine	0.94	[0.75;1.19]	Carboxylic acids and derivatives	Amino acids, peptides, and analogues	Yes	[31]	HMDB0000182

d	Lysine	0.76	[0.53;1.10]	Carboxylic acids and derivatives	Amino acids, peptides, and analogues	Yes	[32]	HMDB0000182
e	Lysine	0.74	[0.48;1.15]	Carboxylic acids and derivatives	Amino acids, peptides, and analogues	Yes	[32]	HMDB0000182
b	Methionine	1.21	[0.88;1.66]	Carboxylic acids and derivatives	Amino acids, peptides, and analogues	Yes	[31]	HMDB0000696
d	Methionine	0.75	[0.54;1.05]	Carboxylic acids and derivatives	Amino acids, peptides, and analogues	Yes	[32]	HMDB0000696
e	Methionine	0.83	[0.63;1.09]	Carboxylic acids and derivatives	Amino acids, peptides, and analogues	Yes	[32]	HMDB0000696
d	Methionine sulfoxide	1.32	[0.68;2.57]	Carboxylic acids and derivatives	Amino acids, peptides, and analogues	Yes	[32]	HMDB0002005
e	Methionine sulfoxide	1.25	[1.02;1.53]	Carboxylic acids and derivatives	Amino acids, peptides, and analogues	Yes	[32]	HMDB0002005

d	N-methyl-L-histidine	1.67	[1.22;2.27]	Carboxylic acids and derivatives	Amino acids, peptides, and analogues	Yes	[32]	HMDB0255176
e	N-methyl-L-histidine	1.48	[1.14;1.92]	Carboxylic acids and derivatives	Amino acids, peptides, and analogues	Yes	[32]	HMDB0255176
b	Ornithine	1.20	[0.91;1.58]	Carboxylic acids and derivatives	Amino acids, peptides, and analogues	Yes	[31]	HMDB0000214
d	Ornithine	1.07	[0.63;1.83]	Carboxylic acids and derivatives	Amino acids, peptides, and analogues	Yes	[32]	HMDB0000214
e	Ornithine	0.93	[0.73;1.18]	Carboxylic acids and derivatives	Amino acids, peptides, and analogues	Yes	[32]	HMDB0000214
c	PGE2	0.89	[0.73;1.08]	Fatty Acyls	Eicosanoids	No	[33]	HMDB0001220
c	PGE2/FA(20:4-w6)	0.90	[0.75;1.08]	NA	NA	No	[33]	NA
c	PGF1a	1.11	[0.90;1.39]	Fatty Acyls	Eicosanoids	No	[33]	HMDB0002685
c	PGF1a/FA(20:3-w3w6)	1.06	[0.91;1.24]	NA	NA	No	[33]	NA
c	PGF2a	0.98	[0.77;1.26]	Fatty Acyls	Eicosanoids	No	[33]	HMDB0001139

c	PGF2a/FA(20:4-w6)	0.98	[0.80;1.20]	NA	NA	No	[33]	NA
b	Phenylalanine	1.80	[1.25;2.59]	Carboxylic acids and derivatives	Amino acids, peptides, and analogues	Yes	[31]	HMDB0000159
d	Phenylalanine	1.14	[0.66;1.95]	Carboxylic acids and derivatives	Amino acids, peptides, and analogues	Yes	[32]	HMDB0000159
e	Phenylalanine	0.94	[0.75;1.17]	Carboxylic acids and derivatives	Amino acids, peptides, and analogues	Yes	[32]	HMDB0000159
d	Phosphoethanolamine	0.78	[0.53;1.14]	Organic phosphoric acids and derivatives	Phosphate esters	Yes	[32]	HMDB0000224
e	Phosphoethanolamine	0.94	[0.76;1.16]	Organic phosphoric acids and derivatives	Phosphate esters	Yes	[32]	HMDB0000224
b	Proline	1.20	[0.91;1.58]	Carboxylic acids and derivatives	Amino acids, peptides, and analogues	Yes	[31]	HMDB0000162
d	Proline	0.84	[0.55;1.30]	Carboxylic acids and derivatives	Amino acids, peptides, and analogues	Yes	[32]	HMDB0000162

e	Proline	0.82	[0.63;1.07]	Carboxylic acids and derivatives	Amino acids, peptides, and analogues	Yes	[32]	HMDB0000162
d	Putrescine	1.26	[0.86;1.84]	Organonitrogen compounds	Amines	Yes	[32]	HMDB0001414
e	Putrescine	1.33	[1.04;1.70]	Organonitrogen compounds	Amines	Yes	[32]	HMDB0001414
d	Sarcosine	1.10	[0.75;1.62]	Carboxylic acids and derivatives	Amino acids, peptides, and analogues	Yes	[32]	HMDB0000271
e	Sarcosine	0.98	[0.83;1.17]	Carboxylic acids and derivatives	Amino acids, peptides, and analogues	Yes	[32]	HMDB0000271
d	SDMA	1.77	[1.27;2.45]	Carboxylic acids and derivatives	Amino acids, peptides, and analogues	Yes	[32]	HMDB0003334
e	SDMA	1.49	[1.17;1.91]	Carboxylic acids and derivatives	Amino acids, peptides, and analogues	Yes	[32]	HMDB0003334
d	SDMA/arginine	1.56	[1.06;2.30]	NA	NA	Yes	[32]	NA
e	SDMA/arginine	1.33	[1.06;1.66]	NA	NA	Yes	[32]	NA

b	Serine	0.99	[0.78;1.27]	Carboxylic acids and derivatives	Amino acids, peptides, and analogues	Yes	[31]	HMDB0000187
d	Serine	0.92	[0.57;1.49]	Carboxylic acids and derivatives	Amino acids, peptides, and analogues	Yes	[32]	HMDB0000187
e	Serine	1.10	[0.73;1.66]	Carboxylic acids and derivatives	Amino acids, peptides, and analogues	Yes	[32]	HMDB0000187
d	Serotonine	0.92	[0.65;1.30]	Indoles and derivatives	Tryptamines and derivatives	Yes	[32]	HMDB0000259
e	Serotonine	0.93	[0.76;1.14]	Indoles and derivatives	Tryptamines and derivatives	Yes	[32]	HMDB0000259
b	Taurine	1.02	[0.75;1.40]	Organic sulfonic acids and derivatives	Organosulfonic acids and derivatives	Yes	[31]	HMDB0000251
d	Taurine	0.65	[0.45;0.92]	Organic sulfonic acids and derivatives	Organosulfonic acids and derivatives	Yes	[32]	HMDB0000251
e	Taurine	0.90	[0.65;1.23]	Organic sulfonic acids and derivatives	Organosulfonic acids and derivatives	Yes	[32]	HMDB0000251

b	Threonine	1.34	[0.97;1.85]	Carboxylic acids and derivatives	Amino acids, peptides, and analogues	Yes	[31]	HMDB0000167
d	Threonine	0.73	[0.49;1.09]	Carboxylic acids and derivatives	Amino acids, peptides, and analogues	Yes	[32]	HMDB0000167
e	Threonine	0.86	[0.66;1.11]	Carboxylic acids and derivatives	Amino acids, peptides, and analogues	Yes	[32]	HMDB0000167
d	Trimethyllysine	1.75	[1.10;2.81]	Carboxylic acids and derivatives	Amino acids, peptides, and analogues	Yes	[32]	HMDB0001325
e	Trimethyllysine	1.14	[0.92;1.42]	Carboxylic acids and derivatives	Amino acids, peptides, and analogues	Yes	[32]	HMDB0001325
b	Tryptophan	0.91	[0.71;1.18]	Indoles and derivatives	Indolyl carboxylic acids and derivatives	Yes	[31]	HMDB0000929
d	Tryptophan	0.72	[0.53;0.97]	Indoles and derivatives	Indolyl carboxylic acids and derivatives	Yes	[32]	HMDB0000929
e	Tryptophan	0.82	[0.64;1.06]	Indoles and derivatives	Indolyl carboxylic acids and derivatives	Yes	[32]	HMDB0000929

c	TXB2	0.87	[0.73;1.04]	Fatty Acyls	Eicosanoids	No	[33]	HMDB0003252
c	TXB2/FA(20:4-w6)	0.87	[0.73;1.04]	NA	NA	No	[33]	NA
b	Tyrosine	1.64	[1.19;2.26]	Carboxylic acids and derivatives	Amino acids, peptides, and analogues	Yes	[31]	HMDB0000158
d	Tyrosine	1.24	[0.78;1.97]	Carboxylic acids and derivatives	Amino acids, peptides, and analogues	Yes	[32]	HMDB0000158
e	Tyrosine	0.93	[0.69;1.25]	Carboxylic acids and derivatives	Amino acids, peptides, and analogues	Yes	[32]	HMDB0000158
b	Urea	1.10	[0.78;1.56]	Organic carbonic acids and derivatives	Ureas	No	[31]	HMDB0000294
b	Valine	2.72	[1.88;3.93]	Carboxylic acids and derivatives	Amino acids, peptides, and analogues	Yes	[31]	HMDB0000883
d	Valine	0.78	[0.52;1.17]	Carboxylic acids and derivatives	Amino acids, peptides, and analogues	Yes	[32]	HMDB0000883

e	Valine	0.66	[0.52;0.84]	Carboxylic acids and derivatives	Amino acids, peptides, and analogues	Yes	[32]	HMDB0000883
d	α-aminobutyric acid	0.81	[0.61;1.08]	Carboxylic acids and derivatives	Amino acids, peptides, and analogues	Yes	[32]	HMDB0000452
e	α-aminobutyric acid	0.86	[0.67;1.09]	Carboxylic acids and derivatives	Amino acids, peptides, and analogues	Yes	[32]	HMDB0000452
d	γ-aminobutyric acid	0.96	[0.57;1.61]	Carboxylic acids and derivatives	Amino acids, peptides, and analogues	Yes	[32]	HMDB0000112
e	γ-aminobutyric acid	0.85	[0.67;1.09]	Carboxylic acids and derivatives	Amino acids, peptides, and analogues	Yes	[32]	HMDB0000112
d	γ-L-glutamyl-L-alanine	0.60	[0.38;0.96]	Carboxylic acids and derivatives	Amino acids, peptides, and analogues	Yes	[32]	HMDB0006248
e	γ-L-glutamyl-L-alanine	0.90	[0.70;1.16]	Carboxylic acids and derivatives	Amino acids, peptides, and analogues	Yes	[32]	HMDB0006248

a: age, sex, body mass index; b: age, sex, history of diabetes, history of hypertension, current smoking, Killip class; c: age, sex, race, enrolment center, ejection fraction, New York Heart Association class, cardiomyopathy etiology, atrial fibrillation, diabetes, hypertension, chronic kidney disease; d: age, sex, race, enrolment center, smoking status, body mass index, ejection

fraction, New York Heart Association class, atrial fibrillation, diabetes, hypertension; e: age, sex, race, enrolment center, smoking status, body mass index, ejection fraction, New York Heart Association class, atrial fibrillation, diabetes, hypertension, chronic kidney disease; BCAA: Branched-chain amino acids; NA: not applicable.

Table S3. Risk of bias within studies (Newcastle-Ottawa Scale)

Reference	Cohort Name	Selection	Comparability	Outcome	Total
[31]	NA	****	**	***	**** ** ***
[32]	PROSE-ICD	***	**	***	*** ** ***
	GRADE	***	**	***	*** ** ***
[33]	PROOSE-ICD	***	**	***	*** ** ***
[34]	NA	***	**	**	*** ** **

Based on NEWCASTLE - OTTAWA QUALITY ASSESSMENT SCALE COHORT STUDIES
Note: A study can be awarded a maximum of one star for each numbered item within the Selection and Outcome categories. A maximum of two stars can be given for Comparability Selection

1) Representativeness of the exposed cohort
    a) truly representative of the average HF patients in the community *
    b) somewhat representative of the average HF patients in the community *
    c) selected group of users e.g. nurses, volunteers
    d) no description of the derivation of the cohort
2) Selection of the non-exposed cohort
    a) drawn from the same community as the exposed cohort *
    b) drawn from a different source
    c) no description of the derivation of the non-exposed cohort
3) Ascertainment of exposure
    a) secure record (e.g. surgical records) *
    b) structured interview *
    c) written self-report
    d) no description
4) Demonstration that outcome of interest was not present at start of study
    a) yes *
    b) no

Comparability

1) Comparability of cohorts on the basis of the design or analysis
    a) study controls for age and sex (select the most important factor) *
    b) study controls for any additional factor *

Outcome

1) Assessment of outcome
    a) independent blind assessment *
    b) record linkage *
    c) self-report
    d) no description
2) Was follow-up long enough for outcomes to occur
    a) yes (minimum median/mean follow-up time of 1 year) *
    b) no
3) Adequacy of follow up of cohorts
    a) complete follow up - all subjects accounted for *
    b) subjects lost to follow up unlikely to introduce bias - small number lost - > 10 % follow up, or description provided of those lost) *
    c) follow up rate < 10% and no description of those lost
    d) no statement

Table S4. Meta-analysis results for each metabolite.

Metabolites	I^2 (%)	p-value for I^2	Combined HR [95% CI]	No. of studies
2-aminoadipic acid	0	0.43	0.91 [0.74;1.10]	2
3-methyl histidine	88.8	<0.01	1.67 [0.94;2.96]	3
ADMA	0	0.83	1.18 [0.89;1.57]	2
ADMA/arginine	0	0.75	1.08 [0.90;1.30]	2
Alanine	56.5	0.10	0.89 [0.69;1.14]	3
Arginine	0	0.67	0.94 [0.79;1.12]	3
Asparagine	0	0.73	1.04 [0.87;1.25]	3
Aspartic acid	0	0.69	0.99 [0.83;1.18]	2
Citrulline	62.0	0.07	1.18 [0.86;1.61]	3
DL-5-hydroxylysine	0	0.97	1.25 [1.05;1.48]	2
Ethanolamine	0	0.38	1.07 [0.85;1.34]	2
Glutamate	0	0.58	1.15 [0.97;1.35]	3
Glutamine	30.4	0.24	0.86 [0.69;1.06]	3
Glycine	0	0.67	1.09 [0.91;1.30]	3
Glycylglycine	0	0.48	1.12 [0.95;1.32]	2
Histidine	0	0.44	0.74 [0.64;0.86]	3
Homoserine	15.1	0.28	0.97 [0.77;1.23]	2
Hydroxyproline	61.2	0.08	1.17 [0.92;1.48]	3
Isoleucine	92.5	<0.01	1.33 [0.66:2.66]	3
Kynurenine	51.4	0.13	1.38 [1.12;1.71]	3
Kynurenine/tryptophan	71.0	0.06	1.66 [1.19;2.31]	2
Leucine	93.2	<0.01	1.14 [0.49;2.67]	3
Lysine	0	0.48	0.86 [0.72;1.03]	3
Methionine	58.6	0.09	0.91 [0.69;1.20]	3
Methionine sulfoxide	0	0.88	1.26 [1.03;1.52]	2
N-methyl-L-histidine	0	0.56	1.56 [1.27;1.90]	2
Ornithine	0	0.39	1.05 [0.86;1.28]	3
Phenylalanine	77.5	0.01	1.23 [0.82;1.86]	3
Phosphoethanolamine	0	0.40	0.90 [0.75;1.08]	2
Proline	52.9	0.12	0.95 [0.73;1.24]	3
Putrescine	0	0.81	1.31 [1.06;1.61]	2
Sarcosine	0	0.59	1.00 [0.85;1.17]	2
SDMA	0	0.41	1.58 [1.30;1.93]	2
SDMA/arginine	0	0.48	1.38 [1.14;1.68]	2
Serine	0	0.85	1.00 [0.83;1.21]	3
Serotonin	0	0.96	0.93 [0.78;1.10]	2
Taurine	44.2	0.17	0.85 [0.66;1.10]	3
Threonine	69.6	0.04	0.95 [0.67;1.34]	3
N-6-Trimethyllysine	62.2	0.10	1.34 [0.89;2.01]	2
Tryptophan	0	0.51	0.82 [0.71;0.96]	3
Tyrosine	69.1	0.04	1.23 [0.86;1.75]	3
Valine	95.1	<0.01	1.11 [0.46;2.67]	3
α-aminobutyric acid	0	0.75	0.84 [0.70:1.01]	2
γ-aminobutyric acid	0	0.68	0.87 [0.70;1.08]	2
γ-L-glutamyl-L-alanine	55.9	0.13	0.77 [0.52;1.13]	2

ADMA: Asymmetric dimethylarginine; SDMA: Symmetric dimethylarginine

Table S5. Prisma 2020 Checklist.

Section and Topic	Item #	Checklist item	Location where item is reported
**TITLE**			
Title	1	Identify the report as a systematic review.	Page 1
**ABSTRACT**			
Abstract	2	See the PRISMA 2020 for Abstracts checklist.	Page 1
**INTRODUCTION**			
Rationale	3	Describe the rationale for the review in the context of existing knowledge.	Page 1. paragraph 1 and 2 and page 2, paragraph 1 to 3, "Heart failure (HF) is a complex clinical syndrome caused by structural and/or functional heart abnormalities, resulting in elevated intracardiac pressures and/or inadequate cardiac output [1]. The prevalence of HF is estimated to be 1-2% of the overall adult population [2], affecting over 10% of those aged over 70 years and with a rising incidence [1]. Patients diagnosed with HF have poor prognosis, with an associated 1-year mortality risk of 15-30% and 1-year hospital readmission risk of 50% [3].<br>Currently, the assessment and monitoring of HF patients consists of the combined evaluation of clinical signs and symptoms, echocardiography and blood natriuretic pep-tides (namely B-type natriuretic peptide – BNP or N-terminal pro-B-type natriuretic pep-tide – NT-proBNP) [1,4], whose levels are known to associate with prognosis [5]. While many other biomarkers have been proposed, such as markers of myocardial injury (cardiac troponins T and I) [6], inflammation and oxidative stress (interleukin-6 and tumor necrosis factor alfa) [7], vascular dysfunction (endothelin-1) [8], and matrix remodeling (matrix metalloproteinase-2) [9], and shown to provide incremental prognostic value over natriuretic peptides, there is no evidence of their incremental benefit in HF management [10,11].<br>Metabolomics is a valuable approach to uncover the molecular processes and bio-logical pathways affected in pathological states, thus contributing to the understanding of complex multifactorial diseases [12], as is the case with HF. Hence, it is a conceivable strategy to explore the onset of metabolic dysregulations underlying CV events [12]. Given the multifactorial nature of HF, often diagnosed in older patients with other underlying comorbidities [1], metabolomics may provide important insights into the disease pathogenesis as well as to identify putative biomarkers potentially associated with patient's clinical trajectories and outcomes [12,13]. The most prominent advances in metabolomics in HF have suggested that HF severity and prognosis may be reflected in the plasma metabolome [14,15]. In fact, a poor prognosis of HF patients has been associated with changes in circulating ceramides, amino acids, acylcarnitines and organic acids, which broadly indicate to a switch in energy and amino acids metabolism [14,15]. Few studies have proposed different metabolite-based profiles such as the Prognostic Metabolic Profile (PMP) or the Cardiac Lipid Panel (CLP) [16,17], the latter showing promising results com-pared to other clinical scores [17].<br>However, none of the metabolite-based scores has reached a clinical application, due to the lack of robust comparison with scores already in clinical use.<br>Despite significant efforts to identify blood metabolites with prognostic value, there is no systematic, quantitative, or qualitative compilation of the current knowledge in the HF population. Therefore, this meta-analysis aimed to gather risk associations between blood metabolites and CV outcomes in patients with HF."
Objectives	4	Provide an explicit statement of the	Page 2, paragraph 3, "Therefore, this meta-analysis aimed to gather risk associations between blood

Section and Topic	Item #	Checklist item	Location where item is reported
		objective(s) or question(s) the review addresses.	metabolites and CV outcomes in patients with HF.”
**METHODS**			
Eligibility criteria	5	Specify the inclusion and exclusion criteria for the review and how studies were grouped for the syntheses.	Page 12, paragraph 10 and page 13, paragraph 1, “In this study, we only included original publications without any language re-strictions. Reviews and meta-analyses, comments, guidelines, editorials or letters, conference summaries, and non-longitudinal studies were excluded. From the results of the initial query, we filtered based on the following inclusion criteria: (1) studies that considered adult (>18 years old) patients diagnosed with HF; (2) studies that leveraged targeted or untargeted metabolomics; (3) studies that used blood samples as plasma or serum; and (4) studies that reported the time-to-event adjusted association between individual metabolites and major outcomes such as all-cause death or all-cause hospitalization. We excluded articles that reported metabolites not measured in at least two cohorts.”
Information sources	6	Specify all databases, registers, websites, organisations, reference lists and other sources searched or consulted to identify studies. Specify the date when each source was last searched or consulted.	Page 12, paragraph 8, “We performed an electronic search of the PubMed and ISI Web of Knowledge data-bases (last searched on December 31st’ 2022) to identify all articles related to the association of individual blood metabolites and CV outcomes in HF patients. The query string “(Metabolomics OR Lipidomics) AND heart failure” (with “Humans” filter in Pubmed) was used for the search. In ISI Web of Knowledge database, the same query string was used with the exclusion of the “Animal”, “Animals”, “Mouse”, “mice”, “rats”, and “review” keywords.”
Search strategy	7	Present the full search strategies for all databases, registers and websites, including any filters and limits used.	Page 12, paragraph 8, “We performed an electronic search of the PubMed and ISI Web of Knowledge data-bases (last searched on December 31st’ 2022) to identify all articles related to the association of individual blood metabolites and CV outcomes in HF patients. The query string “(Metabolomics OR Lipidomics) AND heart failure” (with “Humans” filter in Pubmed) was used for the search. In ISI Web of Knowledge database, the same query string was used with the exclusion of the “Animal”, “Animals”, “Mouse”, “mice”, “rats”, and “review” keywords.”
Selection process	8	Specify the methods used to decide whether a study met the inclusion criteria of the review, including how many reviewers screened each record and each report retrieved, whether they worked independently, and if applicable, details of automation tools used in the process.	Page 13, paragraph 3, “LSN screened all the article titles and abstracts. Disagreements were resolved by consensus among the LSN, SOD, and ASB. Full texts of articles deemed potentially eligible during the initial screening were obtained for further reading. The search and selection processes can be found in the PRISMA flow diagram (Figure 1).”
Data collection process	9	Specify the methods used to collect data from reports, including how many reviewers collected data from each report, whether they worked independently, any processes for obtaining or confirming data from study investigators, and if applicable, details of automation tools used in the process.	Page 13, paragraph 5, “Data were extracted in a standardized form into a Microsoft Excel spreadsheet by LSN and confirmed by SOD.”
Data items	10a	List and define all outcomes for which data were sought. Specify whether all results that were compatible with each	Page 13, paragraph 1, “(..)studies that reported the time-to-event adjusted association between individual metabolites and major outcomes such as all-cause death or all-cause hospitalization.”

Section and Topic	Item #	Checklist item	Location where item is reported
		outcome domain in each study were sought (e.g. for all measures, time points, analyses), and if not, the methods used to decide which results to collect.	
	10b	List and define all other variables for which data were sought (e.g. participant and intervention characteristics, funding sources). Describe any assumptions made about any missing or unclear information.	Page 13 paragraph 5, "Data were extracted in a standardized form into a Microsoft Excel spreadsheet by LSN and confirmed by SOD. Clinical and methodological characteristics were collected from all included studies: study characteristics (author, year), study design, participant characteristics, cohort name, sample size, analytical platform, metabolomics approach (targeted or untargeted), type of blood sample (serum or plasma), mean or median follow-up time, data preprocessing (e.g., log-transformation, standardization), outcomes assessed, and number of events and variables used for adjustment. For each metabolite, adjusted hazard ratios (HR), 95% confidence intervals (95% CI) were retrieved. Metabolite classes and subclasses were also gathered from the Human Metabolome Database and were included in the database [19]."
Study risk of bias assessment	11	Specify the methods used to assess risk of bias in the included studies, including details of the tool(s) used, how many reviewers assessed each study and whether they worked independently, and if applicable, details of automation tools used in the process.	Page 13, paragraph 9, "The quality of observational included studies was assessed using the Newcastle-Ottawa Scale [20], maximum of nine stars (Table S3), by LSN, SOD and FS."
Effect measures	12	Specify for each outcome the effect measure(s) (e.g. risk ratio, mean difference) used in the synthesis or presentation of results.	Page 13, paragraph 5, "For each metabolite, adjusted hazard ratios (HR), 95% confidence intervals (95% CI) were retrieved."
Synthesis methods	13a	Describe the processes used to decide which studies were eligible for each synthesis (e.g. tabulating the study intervention characteristics and comparing against the planned groups for each synthesis (item #5)).	Page 13, paragraph 5 to 7, "Data were extracted in a standardized form into a Microsoft Excel spreadsheet by LSN and confirmed by SOD. Clinical and methodological characteristics were collected from all included studies: study characteristics (author, year), study design, participant characteristics, cohort name, sample size, analytical platform, metabolomics approach (targeted or untargeted), type of blood sample (serum or plasma), mean or median follow-up time, data preprocessing (e.g., log-transformation, standardization), outcomes assessed, and number of events and variables used for adjustment. For each metabolite, adjusted hazard ratios (HR), 95% confidence intervals (95% CI) were retrieved. Metabolite classes and subclasses were also gathered from the Human Metabolome Database and were included in the database [19].<br>4.5 Data aggregation approach<br>Metabolites were included in the meta-analysis if they were measured in at least 2 studies and if the authors explicitly reported that their levels were log-transformed and standardized. The latter step was used to ensure that the HR values were comparable, thus reducing the bias inherent to the experimental characteristics. Given the small number of publications available, no subgroup or sensitivity analyses were performed."
	13b	Describe any methods required to prepare the data for presentation or synthesis, such as handling of missing summary statistics, or data conversions.	Page 13, paragraph 7, "Metabolites were included in the meta-analysis if they were measured in at least 2 studies and if the authors explicitly reported that their levels were log-transformed and standardized. The latter step was used to ensure that the HR values were comparable, thus reducing the bias inherent to the experimental characteristics. Given the small number of publications available, no subgroup or sensitivity

Section and Topic	Item #	Checklist item	Location where item is reported
			analyses were performed.”
	13c	Describe any methods used to tabulate or visually display results of individual studies and syntheses.	Page 13, paragraph 5, “Data were extracted in a standardized form into a Microsoft Excel spreadsheet by LSN and confirmed by SOD. Clinical and methodological characteristics were collected from all included studies: study characteristics (author, year), study design, participant characteristics, cohort name, sample size, analytical platform, metabolomics approach (targeted or untargeted), type of blood sample (serum or plasma), mean or median follow-up time, data preprocessing (e.g., log-transformation, standardization), outcomes assessed, and number of events and variables used for adjustment. For each metabolite, adjusted hazard ratios (HR), 95% confidence intervals (95% CI) were retrieved. Metabolite classes and subclasses were also gathered from the Human Metabolome Database and were included in the database [19].”
	13d	Describe any methods used to synthesize results and provide a rationale for the choice(s). If meta-analysis was performed, describe the model(s), method(s) to identify the presence and extent of statistical heterogeneity, and software package(s) used.	Page 13, paragraph 7, “Metabolites were included in the meta-analysis if they were measured in at least 2 studies and if the authors explicitly reported that their levels were log-transformed and standardized. The latter step was used to ensure that the HR values were comparable, thus reducing the bias inherent to the experimental characteristics. Given the small number of publications available, no subgroup or sensitivity analyses were performed.”<br>And page 13 paragraph 11 to 13, “Continuous variables are shown as means (standard deviations) or medians (inter-quartile ranges), as reported by the authors in the original publications. Meta-analysis was performed using random-effects models to compute combined statistical measures (HR) and 95% confidence intervals (CI). We chose to use the random-effects model, as it incorporates both within- and between-study variance components [21]. Random-effects models account for the variability among study results beyond chance, using the DerSimonian and Laird method. The choice of this model was made built on the clinical and methodo-logical diversity across studies, which could influence the metabolite levels in HF patients.<br>For each meta-analytical measure, the I^2, which measures the percentage of total variation across studies due to heterogeneity rather than chance, was also calculated. Values of I^2 greater than 50% were considered indicative of substantial heterogeneity, while I^2 lower than 50% were considered low to moderate [21].<br>Calculations were carried out in R Statistical Software R [22], version 4.1.12, along with meta package [23], using the metagen() command for combining HR and calculating overall effect and CI.”
	13e	Describe any methods used to explore possible causes of heterogeneity among study results (e.g. subgroup analysis, meta-regression).	Page 13, paragraph 7, “(..) Given the small number of publications available, no subgroup or sensitivity analyses were performed.”
	13f	Describe any sensitivity analyses conducted to assess robustness of the synthesized results.	Page 13, paragraph 7“ (…) Given the small number of publications available, no subgroup or sensitivity analyses were performed.”
Reporting bias assessment	14	Describe any methods used to assess risk of bias due to missing results in a synthesis (arising from reporting biases).	Given the small number of publications available, this analysis was not performed.
Certainty assessment	15	Describe any methods used to assess certainty (or confidence) in the body of evidence for an outcome.	Given the small number of publications available, this analysis was not performed.
**RESULTS**			

Section and Topic	Item #	Checklist item	Location where item is reported
Study selection	16a	Describe the results of the search and selection process, from the number of records identified in the search to the number of studies included in the review, ideally using a flow diagram.	Page 2, paragraph 6, "The flow diagram of the study is shown in Figure 1. In the original search, 718 entries were found, all of which were published from 2005 to 2022, 131 were duplicated. The remaining 587 records were screened by title and abstract, of which 157 were retrieved, and their eligibility was assessed through full-text analysis. Out of these, 7 studies met all the inclusion criteria except for the data preprocessing criteria (log-transformation and standardization) [17,24–29]. One article met all the inclusion criteria, but the metabolite measured was not measured in any other cohort [30]. A total of 4 articles were eligible and sought for meta-analysis (Table 1) [31–34]."
	16b	Cite studies that might appear to meet the inclusion criteria, but which were excluded, and explain why they were excluded.	Page 6, paragraph 10 and 11, "Preprocessing of data is a critical step, impacting the robustness of the findings (i.e., the amount of biologically relevant information within the study) and the comparability of results (between studies) [35]. We defined the use of log-transformation and standardization (to overcome skewness and heteroscedasticity) as an inclusion criterion to ensure the comparability of the HR. Thus, the associations between outcomes and metabolites, in this case HR, refer to a 1-SD change in the log-transformed metabolite range, minimizing differences inherent to the equipment/laboratory used (i.e., sensitivity or limits of detection).<br>We observed a significant dispersion in data preprocessing. Some studies met all the inclusion criteria but refrained from employing any form of pretreatment or transformation [25,26,28,29], whereas others exclusively transformed the metabolite data [17,24,27]. Additionally, one study only pre-processed the data concerning a single metabolite instead of the whole dataset [34]. This lack of standardization in data analysis and reporting significantly limited the number of studies that could be combined through meta-analysis."
Study characteristics	17	Cite each included study and present its characteristics.	Page 4 and 5, Table 1
Risk of bias in studies	18	Present assessments of risk of bias for each included study.	Page 6, paragraph 8 and Table S3, "The Newcastle-Ottawa Scale confirmed the good quality of all included studies (Table S3), with a minimum of 7 [34] and a maximum of 9 stars [31] (maximum range of scale grade is 9)."
Results of individual studies	19	For all outcomes, present, for each study: (a) summary statistics for each group (where appropriate) and (b) an effect estimate and its precision (e.g. confidence/credible interval), ideally using structured tables or plots.	Page 6, paragraph 13, and page 7 paragraph 1 and 2, "Of the 42 metabolites and 3 ratios explored, the meta-analysis showed that 7 metabolites and 1 metabolite ratio were relevantly associated (HR and 95%CI > 1 or < 1, I^2< 50%) with the cardiovascular outcome (Figure 2 and Table S4). Higher histidine (3 studies, pooled HR 0.74, 95%CI [0.64; 0.86], I^2: 0%, p-value = 0.44) and tryptophan (3 studies, pooled HR 0.82 [0.71; 0.96], I^2: 0%, p-value = 0.51) levels were associated with a lower risk of CV events, whereas higher symmetric dimethylarginine (SDMA) (2 studies, pooled HR 1.58 [1.30; 1.93], I^2: 0%, p-value = 0.41), N-methyl-1-histidine (2 studies, pooled HR 1.56 [1.27;1.90] I^2: 0%, p-value = 0.56), SDMA/arginine (2 studies, pooled HR 1.38 [1.14; 1.68], I^2: 0%, p-value = 0.48), putrescine (2 studies, pooled HR 1.31 [1.06; 1.61], I^2: 0%, p-value = 0.81), methionine sulfoxide (2 studies, pooled HR 1.26 [1.03; 1.52], I^2: 0%, p-value = 0.88), and 5-hydroxylysine (2 studies, pooled HR 1.25 [1.05; 1.48], I^2: 0%, p-value = 0.97) levels were associated with a higher risk of CV events. Kynurenine (3 studies, pooled HR 1.38 [1.12;1.71], I^2: 51.4%, p-value = 0.13 and the kynurenine/tryptophan ratio (2 studies, pooled HR 1.66 [1.19;2.31], I^2: 71%, p-value = 0.06) were statistically associated (HR and 95%CI > 1) with the outcome but failed to meet the low to moderate heterogeneity (I^2) criteria.<br>Considering the small number of studies that were pooled, publication bias was not evaluated."
Results of syntheses	20a	For each synthesis, briefly summarise the characteristics and risk of bias among contributing studies.	Page 5 paragraph 3 and page 6, paragraph 1 and 2, "The selected studies included 4 cohorts [31–34], with a total of 1158 patients. The minimal number of patients was 138 [31], and the maximum was 479 [33]. The included studies reported the HR for a total of 83 metabolites and 37 metabolite ratios (Table S2), of which 42 metabolites and 3 ratios were reported in at least two. Follow-up times were re-ported as mean [34], or

Section and Topic	Item #	Checklist item	Location where item is reported
			median [31–33], and ranged from 1 year to [34], to 6.3 years [33] The prevalence of events (calculated as the percentage of events within each N) ranged from 13% [34] to 38% [31].<br>All four cohorts included patients diagnosed with HF, although some differences should be acknowledged (Table 1). Du et al. included patients with acute heart failure (AHF) after primary percutaneous coronary intervention for ST-segment elevation myocardial infarction treatment [31]. Kouzu et al. included both acute and chronic HF patients [34], and all cohorts explored by Zhang et al. included patients with HF with reduced ejection fraction (HFrEF) [32,33]."
	20b	Present results of all statistical syntheses conducted. If meta-analysis was done, present for each the summary estimate and its precision (e.g. confidence/credible interval) and measures of statistical heterogeneity. If comparing groups, describe the direction of the effect.	Page 6, paragraph 13 and page 7 paragraph 1 and 2, "Of the 42 metabolites and 3 ratios explored, the meta-analysis showed that 7 metabolites and 1 metabolite ratio were relevantly associated (HR and 95%CI > 1 or < 1, $I^2 < 50\%$) with the cardiovascular outcome (Figure 2 and Table S4). Higher histidine (3 studies, pooled HR 0.74, 95%CI [0.64; 0.86], I^2: 0%, p-value = 0.44) and tryptophan (3 studies, pooled HR 0.82 [0.71; 0.96], I^2: 0%, p-value = 0.51) levels were associated with a lower risk of CV events, whereas higher symmetric dimethylarginine (SDMA) (2 studies, pooled HR 1.58 [1.30; 1.93], I^2: 0%, p-value = 0.41), N-methyl-1-histidine (2 studies, pooled HR 1.56 [1.27;1.90] I^2: 0%, p-value = 0.56), SDMA/arginine (2 studies, pooled HR 1.38 [1.14; 1.68], I^2: 0%, p-value = 0.48), putrescine (2 studies, pooled HR 1.31 [1.06; 1.61], I^2: 0%, p-value = 0.81), methionine sulfoxide (2 studies, pooled HR 1.26 [1.03; 1.52], I^2: 0%, p-value = 0.88), and 5-hydroxylysine (2 studies, pooled HR 1.25 [1.05; 1.48], I^2: 0%, p-value = 0.97) levels were associated with a higher risk of CV events. Kynurenine (3 studies, pooled HR 1.38 [1.12;1.71], I^2: 51.4%, p-value = 0.13 and the kynurenine/tryptophan ratio (2 studies, pooled HR 1.66 [1.19;2.31], I^2: 71%, p-value = 0.06) were statistically associated (HR and 95%CI > 1) with the outcome but failed to meet the low to moderate heterogeneity (I^2) criteria.
	20c	Present results of all investigations of possible causes of heterogeneity among study results.	NA, *vide supra*
	20d	Present results of all sensitivity analyses conducted to assess the robustness of the synthesized results.	NA, *vide supra*
Reporting biases	21	Present assessments of risk of bias due to missing results (arising from reporting biases) for each synthesis assessed.	NA, *vide supra*
Certainty of evidence	22	Present assessments of certainty (or confidence) in the body of evidence for each outcome assessed.	NA, *vide supra*
**DISCUSSION**			
Discussion	23a	Provide a general interpretation of the results in the context of other evidence.	Page 9, paragraph 3 to page 11, paragraph 4, "Symmetric Dimethylarginine is a derivative of L-arginine generated by the post-translational methylation of arginine residues, such as its isomer, Asymmetric Dimethylarginine (ADMA). This methylation occurs by the action of enzymes from the protein arginine methyltransferases family [38]. Both free ADMA and SDMA are released following proteolysis, although arising from different metabolic pathways, involving protein arginine methyltransferase type 1 and 2 (PRMT1, PRMT2), respectively [39].<br>It has been reported that ADMA, and to a much lower extent SMDA, diminish nitric oxide (NO) bioavailability. ADMA directly inhibits the NO synthase activity [39], and SDMA may interfere with the use

Section and Topic	Item #	Checklist item	Location where item is reported
			of the enzyme substrate L-arginine, which leads to an indirect inhibition [40]. NO plays a vital role in cardiovascular physiology, linked to endothelial function, cardiac contractibility, and cardiac protection [41]. In the context of HF, NO inhibits the chronic β-adrenergic response of ventricular myocardium, which is enhanced in this condition [41]. ADMA and SDMA have been previously shown to be independent markers of all-cause mortality across different types of populations including those with CVD [42]. Bode-Bo et al. established a relationship between high SDMA plasma levels and patients with coronary artery disease [40], while Potočnjak et al. linked high SDMA levels to mortality in acute HF patients [43].<br>3.2. Putrescine<br>Putrescine is produced by ornithine decarboxylation, which acts on a metabolite produced in the breakdown of arginine (Figure 3). This polyamine serves as one of the precursors for other polyamines, including spermidine and spermine [44]. Polyamines are strongly positively charged at physiological pH and bind to acidic sites on cellular macromolecules including proteins, nucleic acids and phospholipid membranes, regulating their activity [45]. Therefore, polyamines regulate several biological processes such as cell division, apoptosis, and gene transcription processes [46], and have been implicated in cardiac hypertrophy in animal models [47]. In humans, a study involving 17 heart failure patients demonstrated an association between the enzymatic activity of ornithine decarboxylation and left atrial hemodynamic overload, along with increased levels of polyamines and improvement in ventricular inotropism [48]. These findings suggest that poly-amine production and subsequent putrescine represent early events in cardiac hypertrophy. Cardiac hypertrophy is an adaptive response to increased functional demand on the heart and may be the result of a large variety of stimuli [49]. Diseases like hypertension and myocardial infarction lead to pathological cardiac hypertrophy which can ultimately induce HF [49]. Given the implications of polyamines, including putrescine, in cardiac hypertrophy, they hold potential as markers for hypertrophy progression and, ultimately, HF.<br>3.3. N-methyl-histidine<br>Protein methylation primarily targets basic amino acid residues like arginine, lysine and histidine [50]. Histidine methylation extends beyond the histone code. This posttranslational modification has been identified by mass spectrometry in non-histone proteins such as actin and myosin [50]. The histidine N-methyltransferase SETD3 targets His73 of actin, methylating this amino acid residue at N3 of the imidazole ring, which has an impact on muscle contractibility [50]. METTL9 catalyzes the methylation of histidine at N1, forming 1-methylhistidine, in the inflammatory protein S100A9 (Figure 3) [51]. Notably, elevated levels of free 1-methylhistidine have already been linked to hypertension [52], diastolic dysfunction [53], and heart failure [54]. These associations may imply changes in the turnover rates of proteins methylated on histidine residues.<br>3.4. Hydroxylysine<br>Hydroxylysine, a hydroxylated derivative of lysine, is present in various types of collagens [55]. This hydroxylation is catalyzed by lysyl hydroxylases and is critical for the following glycosylation and in determining the fate of covalent cross-linking, which contributes to the toughness and resiliency of collagens, thereby influencing their structural properties (Figure 3) [55]. Free forms of hydroxylysine can arise through proteolytic degradation of collagen, and the urinary excretion of 5-Hydroxylysine serves as an index of collagen degradation. Elevated levels of urinary hydroxylysine are indicative of more rapid or extensive collagen degradation [56]. Cardiac collagen remodeling is a crucial step in HF progression, with higher blood levels of its degradation markers being observed in HF populations [57]. Although hydroxylysine is not an exclusive component of heart-derived collagen, it is a key component and a possible marker of collagen synthesis and turnover, especially in states associated with increased myocardial fibrosis,

Section and Topic	Item #	Checklist item	Location where item is reported
			such as HF, suggesting altered collagen pathway degradation in patients with worse prognosis.<br>3.5. Methionine sulfoxide<br>Methionine sulfoxide arises through the oxidation of methionine residues in proteins by reactive oxygen species (ROS) under various physiological or pathological conditions [58]. Its levels are dependent on the redox status in the organ, and on the methionine sulfoxide reductase system that can reduce methionine sulfoxide to methionine (Figure 3). The role of free methionine sulfoxide in the regulation of cellular processes is poorly com-prehended [58]. Nevertheless, because of its connection to ROS, methionine sulfoxide is recognized as a biomarker of oxidative stress in various conditions connected to oxidative stress, for example, aging, type 2 diabetes, chronic renal failure, and ischemic conditions [59,60]. In a cross-sectional analysis of the Bogalusa Heart Study, a population-based study that analyzed the natural course of CVD across the lifespan, methionine sulfoxide was associated with the presence of diastolic dysfunction [53]. The study suggested that methionine sulfoxide could be released from cardiac myocyte protein turnover in the presence of left ventricular diastolic dysfunction or systemic endothelial dysfunction. Furthermore, reduced levels of methionine sulfoxide reductase have been observed during ischemia, possibly implicating this enzyme in vascular disease and cardiac ischemia [60]. In our meta-analysis, methionine sulfoxide was associated with poor outcomes, although its role in HF pathology remains unclear.<br>3.6. Histidine.<br>Histidine is an essential amino acid used in the biosynthesis of proteins [61]. It contains an imidazole functional group capable of scavenging ROS generated during acute inflammatory response [62]. This property imparts histidine with antioxidant and anti-inflammatory characteristics [63]. Liu et al, showed that histidine levels were reduced in HF patients, when compared with healthy controls, demonstrating its sensitivity in distinguishing the two groups [64]. Anguita et al demonstrated that plasma levels of three metabolites, including formate, lactate, and histidine, were determinant for the classification between decompensated or stable heart failure with reduced ejection fraction (HFpEF) [65]. Low histidine levels are associated with poor outcomes, possibly implicating a disruption in inflammatory processes in patients with HF.<br>3.7. Tryptophan<br>Tryptophan is an essential amino acid utilized in protein biosynthesis and is mainly catabolized in pro-inflammatory states [66], generating kynurenine and hydroxykynurenine among others [67]. In previous works, higher levels of tryptophan were found to be associated with lower HF risk, while kynurenine and hydroxykynurenine, showed the opposite trend, plausibly linked to the tryptophan–kynurenine pathway and the inflammatory state in HF patients [68]. Inflammation converts tryptophan into kynurenine and hydroxykynurenine, resulting in reduced levels of tryptophan and in-creased levels of kynurenine and hydroxykynurenine [67]. These two tryptophan catabolites were already linked to HF prognosis [28,34,69], but these findings were not included in our meta-analysis as the studies did not match all the inclusion criteria (lack of the pre-specified data preprocessing)."
	23b	Discuss any limitations of the evidence included in the review.	Page 12, paragraph 4, "The present review and meta-analysis are subject to several limitations, including 1) the lack of studies with comparable and similar data treatment, thereby restricting the range of metabolites that could be included; 2) the existence of heterogeneity in terms of the study population and endpoints; and 3) the absence of a subgroup or sensitivity analysis for the metabolites that were subjected to meta-analysis."
	23c	Discuss any limitations of the review processes used.	Page 12, paragraph 4, "The present review and meta-analysis are subject to several limitations, including 1) the lack of studies with comparable and similar data treatment, thereby restricting the range of metabolites that could be included; 2) the existence of heterogeneity in terms of the study population and endpoints; and

Section and Topic	Item #	Checklist item	Location where item is reported
			3) the absence of a subgroup or sensitivity analysis for the metabolites that were subjected to meta-analysis."
	23d	Discuss implications of the results for practice, policy, and future research.	Page 11 paragraph 6 to page 12 paragraph 2, "Metabolomics is one of the youngest "omics" field to emerge and is considered a promising tool in a clinical scenario [12], as it offers the key advantage of simultaneous measurement of hundreds of metabolites in a single experimental run. As these molecules participate in different biological processes, their change may pinpoint perturbations in specific pathways, making metabolomics valuable for hypothesis-generation studies. Moreover, personalized metabolomics further recognizes the importance of each individual's traits and characteristics, such as comorbidities (e.g. obesity, hypertension, diabetes) and lifestyle (e.g. dietary and exercise habits) in defining their clinical trajectory and out-come. Thus, metabolomics may play an important role in the understanding of the pathophysiology and mechanisms of complex multifactorial diseases, shedding light into new pharmaceutical research towards the identification of novel therapeutic agents.<br>However, despite such huge data sets collected to date, and such promising perspectives of personalized metabolomics, there are yet no clinical metabolomics application [70]. One of the main problems discussed in the literature and corroborated in this study is the lack of standardization across all stages of research. Only through the standardization of analytical strategies, data statistical analysis and reporting, findings may be compared and combined [70]. The Metabolomics Standards Initiative (MSI) [71] and the Framework Programme 7 EU Initiative 'coordination of standards in metabolomics' (COSMOS) [72] have already pinpointed the importance of standardization in all stages of the metabolomic framework. The metabolomic community would greatly benefit from having standard protocols including all analytical steps (sample collection, handling and analysis), data handling (pre-processing and statistical modeling). Not less important is the need for data sharing standards, for instance through the use of checklists for data and metadata [71] that ensures compliance with FAIR principles, are key to envisage adequate exchange, comparison and re-utilization of metabolomics datasets [72]. Another significant draw-back, although with few exceptions in literature, is the lack of reporting standards, with most metabolomics studies presenting their findings in non-quantitative scales (i.e. log-transformed, standardized or normalized to total intensities) instead of quantitative ones (mg/dL or mmol/dL), impacting the potential clinical applications of such findings."
**OTHER INFORMATION**			
Registration and protocol	24a	Provide registration information for the review, including register name and registration number, or state that the review was not registered.	Page 12, paragraph 6, "This review protocol was not registered in any platform
	24b	Indicate where the review protocol can be accessed, or state that a protocol was not prepared.	Page 12, paragraph 6, "This review protocol was not registered in any platform."
	24c	Describe and explain any amendments to information provided at registration or in the protocol.	Page 12, paragraph 6, "This review protocol was not registered in any platform."
Support	25	Describe sources of financial or non-financial support for the review, and the role of the funders or sponsors in the review.	Page 14, paragraph 5 "**Funding:** FCT - Portuguese Foundation for Science and Technology, under the scope of the Cardiovascular R&D Center – UnIC (UIDB/00051/2020 and UIDP/00051/2020) and RISE (LA/P/0053/2020), and CardioNIR: CARDIOvascular Near-InfraRed spectroscopy probing", ref: PTDC/EMD-EMD/3822/2021 (https://doi.org/10.54499/PTDC/EMD-EMD/3822/2021)."
Competing interests	26	Declare any competing interests of	Page 14, paragraph 7 "**Conflicts of Interest:** The authors declare no conflicts of interest."

Section and Topic	Item #	Checklist item	Location where item is reported
		review authors.	
Availability of data, code and other materials	27	Report which of the following are publicly available and where they can be found: template data collection forms; data extracted from included studies; data used for all analyses; analytic code; any other materials used in the review.	Page 14, paragraph 6, “Data Availability Statement: All data used in this article is available in supplementary materials.”
